# Mapping the Scientific Literature on Sheep and Goat Research: General Appraisal and Significance of the Year of Publication

**DOI:** 10.3390/ani16081163

**Published:** 2026-04-10

**Authors:** Georgia A. Vaitsi, Maria V. Bourganou, Charalambia K. Michael, Natalia G. C. Vasileiou, Eleni I. Katsarou, Angeliki I. Katsafadou, Dimitris A. Gougoulis, Vasia S. Mavrogianni, George C. Fthenakis

**Affiliations:** 1 Veterinary Faculty, University of Thessaly, 43100 Karditsa, Greece; gvaitsi@vet.uth.gr (G.A.V.); elekatsarou@vet.uth.gr (E.I.K.); dgoug@vet.uth.gr (D.A.G.); 2 Faculty of Public and One Health, University of Thessaly, 43100 Karditsa, Greece; mbourganou@uth.gr (M.V.B.); agkatsaf@uth.gr (A.I.K.); 3 School of Veterinary Medicine, European University of Cyprus, Nicosia 2404, Cyprus; cha.michael@euc.ac.cy; 4 Faculty of Animal Science, University of Thessaly, 41110 Larissa, Greece

**Keywords:** animal science, bibliometric, bibliometric analysis, meta-analysis, One Health, review, scientometric, small ruminant, veterinary science

## Abstract

The objectives were: (i) mapping of bibliometric characteristics of publications related to sheep and goats internationally, (ii) comparison of publications related to each animal species, and (iii) comparison of characteristics in a 55-year long timespan from 1970 to 2024. The Web of Science was used with the following search terms: [sheep OR ovine OR *Ovis aries*] or [goat* OR caprine OR *Capra hircus*].account, and 165,052 papers related to sheep and 67,637 papers related to goats were considered. There was a progressive increase in papers published annually, with a higher proportion of papers related to goats published recently. Most papers were published in *Small Ruminant Research* (2.2% and 4.4% of papers related to sheep and goats, respectively), the journal with most published papers for 21 (sheep) and 29 (goats) years. Most papers originated from the United States of America, the country with most published papers for 52 (sheep) and 41 (goats) years. Most published papers related to sheep or goats were classified in the Dairy and animal sciences topics-meso. The two predominant topics-micro were Ruminant nutrition and Livestock reproduction for published papers related to sheep and papers related to goats. Overall, 31.7% and 34.9% of papers related to sheep and goats, respectively, were published under open access, with a progressive increase yearly. On average, papers related to sheep had received 0.93 citations annually and papers related to goats 0.73 citations annually.

## Simple Summary

The present work has focused on mapping of published papers related to sheep and goats by means of bibliometric analysis. The work provided an extensive description and analysis of the scientific output related to sheep and goat research internationally. Work on both species shows strong thematic stability. However, changes have been detected in the origin of the published papers in recent years, as well as in the journals used for dissemination of the results. A trend emerges that potentially in the future, new fields of work can develop, such as, for example, climate adaptation research.

## 1. Introduction

Mapping of the output of scientific research helps to analyze, characterize and present relevant complex information and allows researchers to determine associations between ideas and to identify research gaps in the international body of scientific knowledge [1,2,3]. Data from bibliometric mapping can be valuable, in order to identify trends and impact in a given discipline through the study of relevant scientific publications, as well to identify the cognitive structure and internal dynamics of these disciplines [1,2]. Connections and relations can emerge between publications, themes and scientists, and, that way, patterns which could not be observed otherwise from a text alone can be revealed. In such studies, the raw and complex data in the publications and reports of results are condensed and categorized and finally presented under more easily understandable formats. Further, through the longitudinal analysis of data, the evolution of a research area over time becomes obvious [1,4,5]. Also, the results of mapping contribute to minimizing bias and presenting a reliable and evidence-based summary of the situation [6].

Scientists and clinicians can benefit in various ways from such works. Mapping of research can guide them to understand the depth and the breadth of their field of work [7]; thus, they can plan strategically their future work pathways. Additionally, the results often reveal influential institutions and laboratories, as well as key actors in the study themes. All these can influence decisions for potential collaborators or tutors in future steps, which may support important decisions [8].

The objectives of the present work were: (i) mapping of bibliometric characteristics of publications related to sheep and goat studies internationally, (ii) comparison of publications related to each of the two animal species and (iii) comparison of main characteristics in a 55-year long timespan from 1970 to 2024.

## 2. Methodological Strategy

The Web of Science platform (www.webofknowledge.com; Clarivate Analytics, London, UK) was employed in this work for the search and retrieval of relevant publications, which consisted of the body of documents for evaluation and analysis [2]. The Web of Science Core Collection was used in the search. The enquiries (full topical searches) were performed by the following strings: (i) {TOPIC = [sheep OR ovine OR *Ovis aries*] *AND* YEAR PUBLISHED = [1970–2024]} for papers related to sheep, and (ii) {TOPIC = [goat* OR caprine OR *Capra hircus*] *AND* YEAR PUBLISHED = [1970–2024]} for papers related to goats. The search was undertaken on 12 September 2025 (‘freeze date’), with an update performed on 22 December 2025. The total time span covered in the study was 55 years.

Then, an analysis of the retrieved body of documents was performed. First, the final date of publication was confirmed and any documents published after the 31 December 2024 were excluded. Thus, only documents published up to the end of 2024 were maintained for further analysis. Thereafter, the types of documents were evaluated and documents other than ‘Articles’ or ‘Reviews’ were excluded. All data were systematically recorded and organized using Microsoft Excel (2019 and 2024) (Microsoft Corporation, Redmond, WA, USA). Subsequently, the following descriptors were obtained and considered [2] for these published papers.

Year of publication of the papers;
(a) Journal in which the papers were published, and (b) Web of Science categories into which each journal was classified;
Language in which the published papers were written;
(a) Country of origin of the published papers and (b) organization of affiliation of the papers;
(a) Topics-meso and (b) topics-micro, as developed by Clarivate Analytics [9], referred to in the published papers;
Accessibility of the published papers under subscription-only or open access;
Sustainable development goals (as defined in the 2030 Agenda for Sustainable Development, adopted by the United Nations [10]), served in the published papers;
Citations received by the published papers; citations were normalized by considering the year of publication; thus, citations received annually were calculated;
Authors of the published papers.

Subsequently, similar searches were performed for other seven species of domestic mammals: buffaloes, camels, cats, cattle, dogs, donkeys, horses, pigs and rabbits (strings detailed in Table S1). Again, only records published between 1970 and 2024 were taken into account and, also, types of documents other than ‘Articles’ or ‘Reviews’ were excluded.

All the data were systematically recorded and organized using Microsoft Excel (versions 2503–2601). Descriptive analyses were made initially. Linear regression analysis was carried out, in order to study associations with the year of publication of the papers. The frequency of the various outcomes was evaluated in tables of cross-categorized frequency data using the Fisher exact test or the Pearson chi-square test. Comparisons between continuous data were performed using the Mann–Whitney test or the Kruskal–Wallis test.

## 3. Results

In total, 185,544 documents related to sheep and 74,532 documents related to goats were retrieved. Overall, 36 types of documents related to sheep and 33 types related to goats were retrieved (Table S2). Among these, there were 165,052 published papers (159,520 (96.6%) articles and 5533 (3.4%) reviews) related to sheep and 67,637 published papers (65,104 (96.3%) articles and 2533 (3.7%) reviews) related to goats (Table S3).

When compared with other domestic mammal species in accord with the number of published papers related to each, sheep were ranked sixth and goats were ranked eighth species (Figure S1).

### 3.1. Year of Publication and Proportion of Papers Published over the Study Timespan

**Figure 1:**
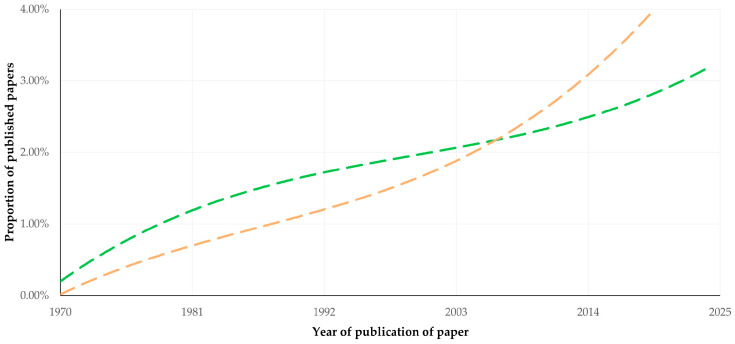
Trendlines for the annual proportion of published papers among all papers related to sheep (green) or to goats (coral) published from 1970 to 2024.

There was a clear progressive increase in the number of published papers annually, which consequently led to a significant difference in the slope of increase in the proportion of papers published annually. A higher proportion of papers related to goats was published in more recent years (slope for published papers related to sheep: 0.0005 ± 0.0001, slope for published papers related to goats: 0.0008 ± 0.0001) (Figure 1, Table S4).

### 3.2. Journals in Which the Papers Were Published

In total, the papers were published in 8239 or 5862 journals for papers related to sheep or to goats, respectively (Table S5). The journal in which most papers related to both sheep and goats were published was *Small Ruminant Research*. However, a smaller proportion of papers related to sheep than papers related to goats was published in that journal: 2.2% versus 4.4%, respectively (Table 1). There were also differences in the proportions of papers related to sheep or to goats published in the various journals with most published papers (Figure S2).

The numbers of years during which the journals were among the top ten scientific journals throughout the study period are in Figure S3. All in all, there were 85 (1.0%) and 150 (1.7%) journals for publication of papers related to sheep or to goats, respectively, among the top ten journals on annual basis. *Small Ruminant Research* was the top journal (i.e., the one with most papers published therein) for 21 (papers related to sheep) and 29 (papers related to goats) years (Table S6).

During the period from 1970 to 1980, these included in total 34 and 83 journals for papers related to sheep and goats, respectively; during the period from 2014 to 2024, these included 28 and 30 different journals, respectively (Appendix A, Table S7). One journal for papers related to sheep (specifically, *Journal of Animal Science*) and four journals for papers related to goats (specifically, *Indian Journal of Animals Sciences*, *Journal of Dairy Science*, *Tropical Animal Health and Production* and *Veterinary Parasitology*) were among these journals during both periods.

### 3.3. Web of Science Categories of Journals in Which the Papers Were Published

In total, the journals were classified into 252 or 251 Web of Science categories for papers related to sheep or to goats, respectively (Table S8). These categories represented all 21 groups of Web of Science categories available in the database. The two categories of journals, in which most papers related to both sheep and goats were published, were ‘Veterinary Sciences’ and ‘Agriculture, Dairy Animal Science’. However, a significantly smaller proportion of papers related to sheep than to goats were included into each of these two categories as follows: 22.6% and 16.1% versus 27.8% and 22.5%, respectively (Table 2, Figure S4). There were also differences in the proportions of papers related to sheep or to goats published in various categories.

**Figure 2:**
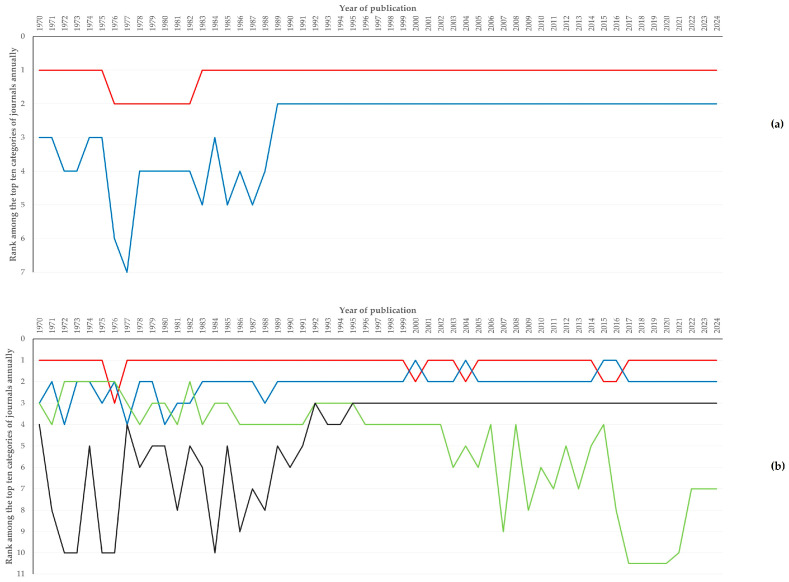
Line plot of the rank among the top categories of journals annually from 1970 to 2024 for papers related to sheep (**a**) or goats (**b**) (each line represents the classification of respective categories annually throughout the study period, with the following colour codes: red line: Veterinary Sciences, blue line: Agriculture Dairy Animal Science, green line: Biochemistry Molecular Biology, black line: Food Science Technology).

The number of years during which the categories of journals were among the top ten categories throughout the study period are in Figure S5. Cumulatively, there were 28 (11.1%) and 31 (12.3%) categories of journals for publication of papers related to sheep or to goats, respectively, included among the top ten categories on annual basis. Veterinary Sciences was the top category for 48 (papers related to sheep) and 50 (papers related to goats) years (Table S9, Figure 2).

During the period from 1970 to 1980, these included 16 and 22 different categories for papers related to sheep and goats, respectively; during the period from 2014 to 2024, these included 14 and 17 different categories, respectively (Appendix B, Table S10).

### 3.4. Languages of Published Papers

In the great majority, the published papers were written in English: 95.1% of papers related to sheep and 94.8% of papers related to goats. Other frequently used languages were German (1.3% and 1.1% of papers, respectively), French (1.0% and 1.1%, respectively) and Portuguese (0.9% and 1.3%, respectively). Overall, 35 different languages were used in the published papers (Table S11).

**Figure 3:**
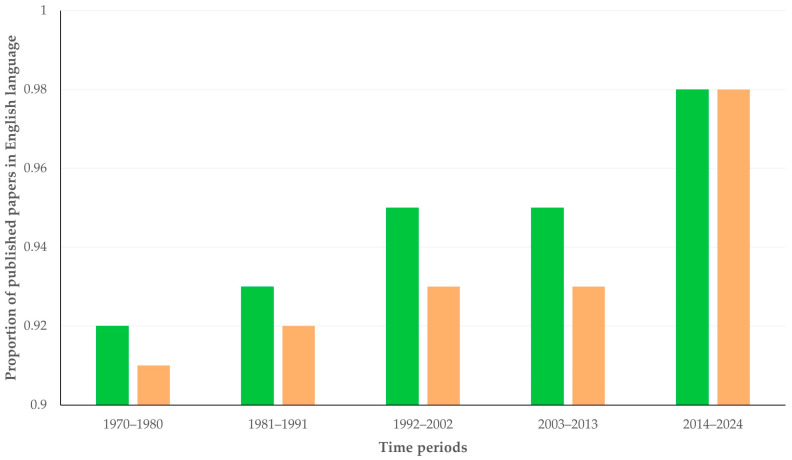
Column plot of the proportion of papers related to sheep (green) or to goats (coral) in English language, in accordance with the time period of publication.

There was a clear progressive increase in the proportion of published papers written in English: from 91.9% (papers related to sheep) and 91.2% (papers related to goats) during the period from 1970 to 1980, to 97.6% and 97.5%, respectively, during the period from 2014 to 2024 (Figure 3).

### 3.5. Countries of Origin of Published Papers

**Figure 4:**
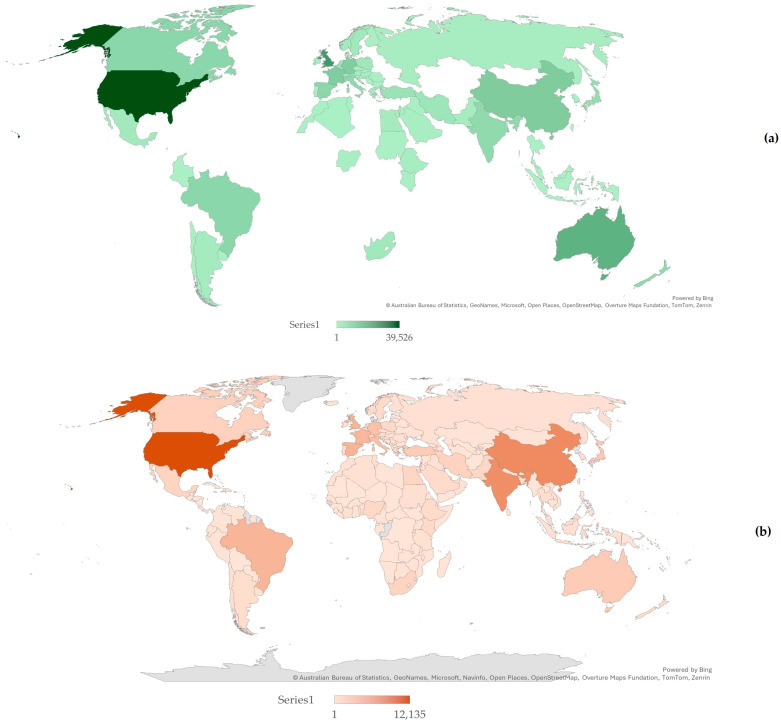
Filled maps of countries in accordance with total number of papers related to sheep (**a**) or to goats (**b**) published from 1970 to 2024.

Papers related to sheep originated from 183 countries, whilst papers related to goats originated from 180 countries (Figure 4) (combined, in total, from 186 countries) (Table S12). For both categories of published papers, most originated from the United States of America (*n* = 39,526 and 12,135, respectively); fewer papers related to sheep originated from the United Kingdom (*n* = 21,304) or Australia (*n* = 15,606), whilst fewer papers related to goats originated from China (*n* = 7280) or India (*n* = 6944) (Figure S6). There were differences in the proportions of published papers related to sheep or to goats that originated from the various countries of the world.

**Figure 5:**
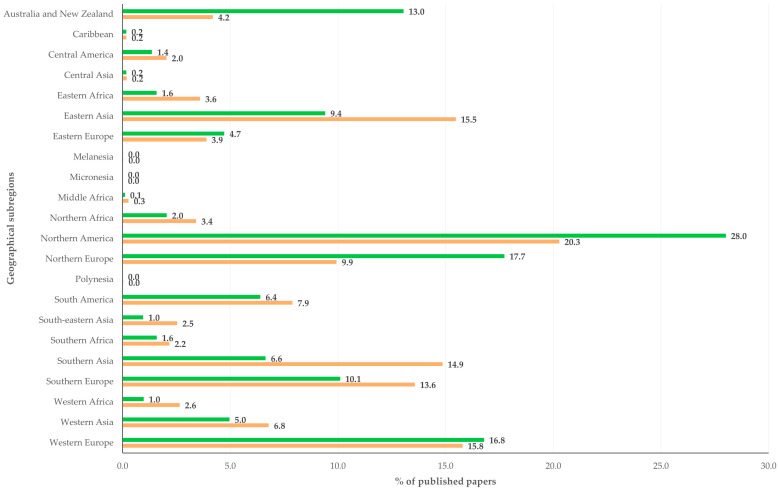
Proportions of papers related to sheep (green) or to goats (coral) published from 1970 to 2024, in accordance with the geographical subregion of the countries of origin.

With regard to the origin of the published papers based on geographical subregions, there were significant differences in the proportions of published papers related to sheep or to goats that originated from the various countries of the world. Most papers related to sheep originated from Northern America, Northern Europe and Western Europe; most papers related to goats originated from Northern America, Western Europe and Eastern Asia (Table S13). The proportion of papers related to sheep was higher than that of papers related to goats in the following five geographical subregions (Australia and New Zealand, Northern Europe, Northern America, Western Europe and Eastern Europe); the proportion of papers related to goats was higher than that of papers related to sheep in the other 17 geographical subregions and particularly in Southern Asia, Eastern Asia and Southern Europe (Figure 5).

The number of years during which countries of origin were among the top ten throughout the study period are in Figure S7. There were 24 (13.1%) and 30 (16.7%) countries of origin for published papers related to sheep or to goats, respectively, among the top ten countries of origin on annual basis. United States of America was the top country of origin for 52 (papers related to sheep) and 41 (papers related to goats) years (Table S14).

During the period from 1970 to 1980, there were in total 17 and 21 countries among the top ten countries annually for papers related to sheep and goats, respectively; during the period from 2014 to 2024 there were 13 and 14 different countries of origin, respectively (Appendix C, Table S15). Six countries (Australia, France, Germany, India, United Kingdom, and United States of America) were among the top ten countries in both periods for papers related to sheep and to goats; moreover, another two countries (Italy, New Zealand) were also included for papers related to sheep.

### 3.6. Organizations of Affiliation in Published Papers

There were 40,199 and 24,327 organizations of affiliations listed in published papers related to sheep and goats, respectively. The French National Institute for Research in Agriculture, Food and Environment and the National Centre for Scientific Research, the Indian Council of Agricultural Research and the United States Department of Agriculture were among the ten scientific organizations with most papers related to each of the two species (Table S16). Among the 100 organizations with most published papers, most were universities: 74% and 76% for published papers related to sheep and goats, respectively, whilst 26% and 24% of published papers originated from other types of organizations (e.g., research institutes, government units, etc.). The majority of the 100 organizations with most published papers related to sheep were in the United States of America (*n* = 22), the United Kingdom (*n* = 15) and Australia (*n* = 13); respectively, the majority with most published papers related to goats were in the United States of America (*n* = 14), Brazil and France (*n* = 10 in each) (Table S17).

### 3.7. Topics-Meso in Published Papers

Papers on sheep were classified in 305 and papers on goats on 307 different topics-meso. Most published papers related to sheep or to goats were classified in the Dairy and animal sciences topics-meso (*n* = 33,137 (20.1%) and 13,436 (19.9%), respectively) (Table 3 and Table S18). There were differences in the proportions of published papers related to sheep or to goats with regard to the topics-meso in the papers.

### 3.8. Topics-Micro in Published Papers

Papers on sheep were classified in 1760 and papers on goats on 1573 different topics-micro. The two predominant topics-micro were Ruminant nutrition and Livestock reproduction for published papers related to sheep and to goats: 15,361 (9.3%) and 8681 (5.2%) versus 5694 (8.4%) and 3377 (5.0%), respectively (Table 4 and Table S19). There were differences in the proportions of published papers related to sheep or to goats with regard to the topics-micro in the papers.

**Figure 6:**
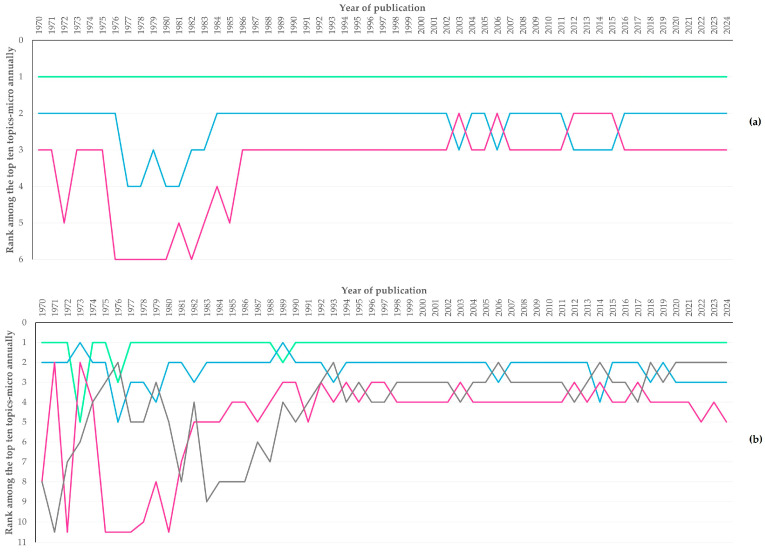
Line plot of the rank among the top topics-micro annually from 1970 to 2024 for papers related to sheep (**a**) or goats (**b**) (each line represents the classification of respective topics-micro annually throughout the study period, with the following colour codes: green line: Ruminant nutrition, blue line: Livestock reproduction, purple line: Anthelmintic resistance, grey line: Dairy product chemistry).

The number of years during which the topics-micro were among the top ten throughout the study period are in Figure S8. There were 43 (2.4%) and 66 (4.2%) topics-micro for published papers related to sheep or to goats, respectively, included throughout this period among the top ten on annual basis. Ruminant nutrition was in top for 55 (papers related to sheep) and 53 (papers related to goats) years during the study period (Table 5, Figure 6).

During the period from 1970 to 1980, these included 25 and 55 different topics-micro for papers related to sheep and goats, respectively; during the period from 2014 to 2024 these included 18 and 19 different topics-micro, respectively (Appendix D, Table S20). Six topics-micro (Anthelmintic resistance, Livestock reproduction, Meat quality, Farm animal welfare, Ruminant nutrition and *Toxoplasma gondii*) were among the top ten during both periods for papers related to sheep and to goats; another one (Embryo development) was among the top ten for papers related to sheep and another eight (Brucellosis, Dairy product chemistry, Mastitis, Male fertility, *Poxvirus* immunology, Tick-borne pathogens, Viral disease dynamics, and Wildlife ecology) were among the top ten for papers related to goats.

### 3.9. Accessibility of Published Papers

**Figure 7:**
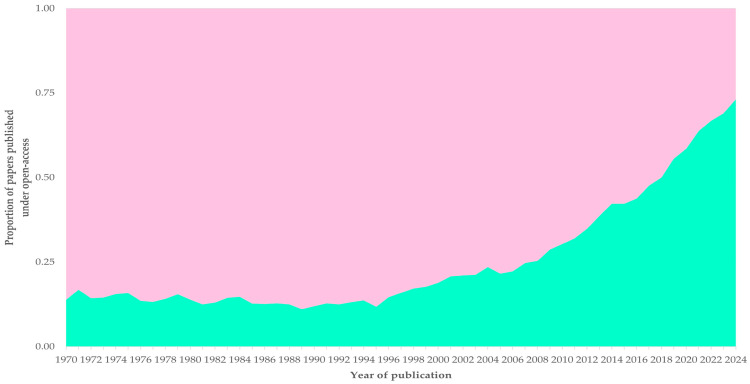
Stacked area plot of the yearly proportion of papers related to sheep or goats published from 1970 to 2004, in accord with type of accessibility, i.e., published under subscription-only (pink) or open (green) access.

Overall, approximately one-third of all published papers was published under open access: 31.7% (*n* = 52,349) of papers related to sheep and 34.9% (*n* = 23,585) of papers related to goats. There was a clearly significant overall increase in the proportion of papers published under open access annually, throughout the study period (1970–2024); slopes were as follows: 0.009 ± 0.001 overall, 0.009 ± 0.001 for papers related to sheep and 0.008 ± 0.001 for papers related to goats (Figure 7 and Figure S9).

During the period from 1970 to 1999, there was no change in the proportion of papers published under open access: slope was −0.001 ± 0.001. An increase in the proportion of papers published under open access was seen from 2000 and thereafter until 2024: slope was 0.023 ± 0.001 with a clear difference between the two periods (Figure 7).

### 3.10. Sustainable Development Goals in Published Papers

For papers related to sheep and papers related to goats, the primary sustainable goals were ‘Good health and well-being’ (70.3% and 67.1% of all papers, respectively) and ‘Climate action’ (14.5% and 13.5% of all papers, respectively). However, there were differences in the proportions of published papers related to sheep or to goats with regard to the sustainable development goals served in the papers (Table S21).

**Figure 8:**
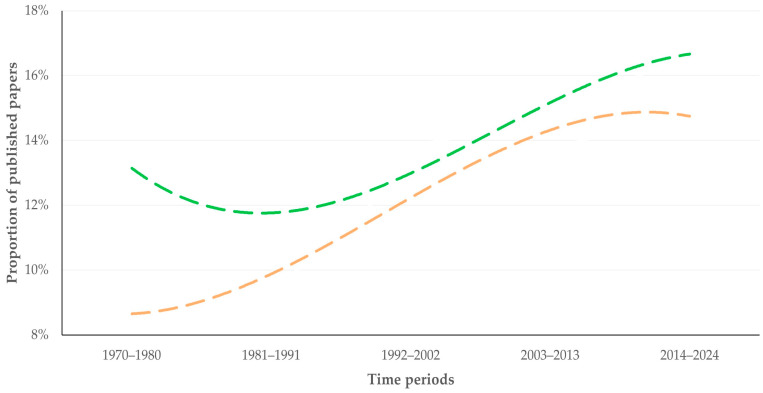
Trendlines for the proportion of published papers that served the ‘Climate action’ sustainable goal among all papers related to sheep (green) or goats (coral) published during each of five time periods from 1970 to 2024.

For papers related to sheep and papers related to goats, ‘Good health and well being’ was the primary goal throughout the study period (>65.0% of papers published within each time period), but with a trend of declining proportion (Figure S10). In contrast, there was a trend for increase in the ‘Climate action’ for papers related to sheep (from 13.3% to 16.8%; slope: 0.001 ± 0.004) and to goats (from 8.8% to 14.9% slope: 0.002 ± 0.003) (Figure 8).

### 3.11. Citations Received by Published Papers

Up to the end of 2024, published papers related to sheep had received a total of 4,211,370 citations. On average, each published paper had received 25.5 citations in total and 0.93 citations annually; of these papers, 37 had received over 1000 citations by the end of 2024. Published papers related to goats had received a total of 1,356,273 citations. On average, each published paper had received 20.1 citations in total and 0.73 citations annually; of these papers, 12 had received over 1000 citations by the end of 2024.

Finally, the *h*-index for the papers related to sheep was 393 and the *h*-index for the papers related to goats was 259.

**Figure 9:**
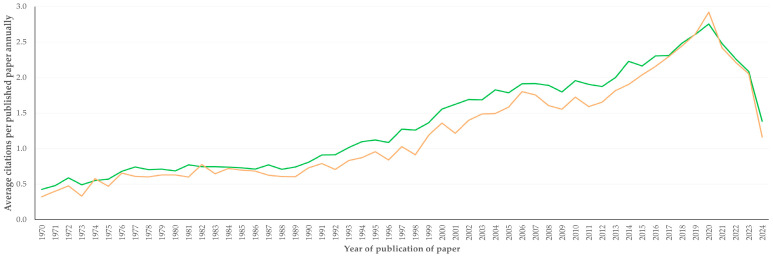
Line plot of average number of citations received annually per paper related to sheep (green) or to goats (coral) published from 1970 to 2024.

There was a clear progressive increase in citations received annually per paper, which was significant: slopes were 0.040 ± 0.002 and 0.039 ± 0.002, for papers related to sheep and goats, respectively, but with no difference seen between papers related to sheep and papers related to goats (Figure 9). Maximum citations received annually were achieved by papers published during the years 2018, 2019, 2020 and 2021: ≥2.4 citations per paper annually (versus an overall average of 0.9 and 0.7 citations annually for papers related to sheep and goats, respectively, i.e., +170% for papers related to sheep and +230% for papers related to goats). Subsequently to 2021, there was a decrease in citations received annually by published papers (Figure 9).

### 3.12. Authors of Published Papers

The ten authors with most published papers related to sheep were distinct to the ten authors with most published papers related to goats. The median numbers of papers published by these authors were 213.5 (interquartile range (IQR): 56) and 132.5 (IQR: 22.5), respectively. Moreover, papers by the former authors have received more citations than papers by the latter ones: 7935.5 (IQR: 5645) versus 2766.5 (IQR: 1183), respectively. Further details about the characteristics of the papers published by these 20 authors are in Table 6. Among these authors, for papers related to sheep, most were based in the United States of America (*n* = 5) and fewer ones in Australia or New Zealand (*n* = 2 in each) and Greece (*n* = 1); for papers related to goats, most authors were based in China (*n* = 4) and fewer in Brazil or the United States of America (*n* = 2 in each) and in France or India (*n* = 1 in each). There were several common papers among these authors (Figure S11); their proportion among the total papers published by each of these authors was lower for papers related to sheep than for papers related to goats: 14.0% versus 17.4%.

## 4. Discussion

### 4.1. Progressive Increase in Published Papers

The clearly evident progressive increase recorded in the published papers throughout the study period, which covers a 55-year long span, is in line with the rapid growth of scientific output observed internationally [11]. This reflects the growth and the evolution of the respective scientific field, for which important determinants have included the progress in scientific and technological advancements worldwide (for example, improvements in methodological possibilities and development of new tools for use in research work), the availability of research funding, the demand for innovation, the expansion of publishing under open access mode publishing and the development of new scientific journals. The increased scientific output has led to the significant improvements in farming and health management of small ruminants, which have dominated the field during these years and resulted in the increase in production yields (meat, milk, wool, and pelts) from these species. These improvements in farming and health management have also coincided with the reshaping of veterinary and animal science at research and clinical level.

At the other end, it should also be noted that the establishment of scientific publications as a key determinant of academic appointments and promotions, and the resulting pressure for career development, has led to increasing the number of scientific outputs from the same piece of research [12,13,14]. This has also contributed to the increasing number of published papers.

The notably higher recent increase in published papers related to goats indicates a clear change in the interest of scientists in that species. Goats had been previously considered with similarities to sheep, which might have limited specific research specifically relevant to that species. However, in contrast to that perception, goats have fundamental differences to sheep, which are reflected in different management and health problems [15,16,17,18,19,20]. As this has become evident, research specifically relevant to goats has been instigated and performed, which has been reflected in the more lifted slope of increase in published papers related to those animals.

This increased research output also reflects the growing importance of these animals in countries outside the United States and Europe [21]. In China and India, the goat sector has been recognized as an important and rapidly growing part of the rural economy, shifting towards intensification and commercialization [21,22,23,24,25,26]. Moreover, goats have been recognized as preferred animals for use in smallholder farming systems [27]. Finally, some of these published papers may reflect an increased interest in goats as a climate-resilient livestock species, which aligns with the facts that goats are often farmed under extensive or semi-extensive management [28] and that the area of environmental sciences has seen a rapid development of research output since 2015 [29].

### 4.2. Journals in Which the Papers Were Published—WoS Categories of Journals

The journals in which the papers were published, as well as the categories in which these journals are classified, can provide a strong indication regarding the content of the papers [30]. The journal dynamics throughout the study period can also provide information regarding changes and shifts in the scientific identity of the published papers.

*Small Ruminant Research* has been a journal dedicated specifically to the promotion and dissemination of work related to sheep and goats since its foundation in 1988 [31]. It has been the dominant journal in the field from the 1990s to 2019; thereafter, *Animals* has taken over as the top journal for papers related to both species. For these two journals, the proportion of papers published during the last time period (2014–2024) has decreased and increased, respectively. The journal *Animals* also offers open access publication as standard, and, moreover, it is classified within the first quartile (Q1) in the relevant WoS categories [32]; in contrast, *Small Ruminant Research* is classified within the second quartile (Q2) therein [31]. We believe that these attributes may play a role in selections made by authors for submission of manuscripts. Indeed, the impact factor of journals has been cited as a variable determining author choices for selecting journals for manuscript submission [33,34]. We also hypothesise that the faster time required for a decision after review (50.0 days for *Small Ruminant Research* versus 17.5 days for *Animals* (as quoted in the webpages of the respective journals [31,32])) might have also contributed towards this significant change, which can be useful when meeting deadlines relevant to dissemination of results.

The findings indicate that papers related to sheep cover topics referring to that species as livestock animals and also as biomedical models. In this latter aspect, the predominant areas of work include endocrinology and reproductive biology, which are considered as foundation areas for human obstetrics and gynecology [35,36]. Indeed, sheep have been widely employed as animal models in the study of human reproduction and pregnancy, due to similarities in the structure of placenta and in development of the fetus(es); additionally, sheep are deemed to be superior to the commonly employed animal models in biomedical research (rodents), because they bear mostly single or twin fetuses, which compares well with human pregnancies [37,38,39,40,41]. This becomes evident when considering the journals, in which papers related to sheep have been published, for example, *Endocrinology*, *Biology of Reproduction*, as well as the predominant categories of journals, for example, Reproductive Biology, Endocrinology Metabolism, and Physiology.

In contrast, papers related to goats have a greater focus towards production and food technology hygiene than papers related to sheep. These papers have remained more focused and were published in journals within the remit of veterinary and animal sciences.

### 4.3. Languages of Published Papers

The significant increase in the proportion of papers published in the English language throughout the study reflects the progressive globalization of scientific research and shows the establishment of English as the international language of science [42,43]. In this respect, one should also take into account that the development of large language models, which may be used by authors in the writing of scientific papers, promote the use of English language [44], which may further contribute to the increase in numbers of papers in English.

While use of English language improves dissemination of research and promotes the potential for collaborations between scientists from various parts of the world, it should also be noted the possibility for marginalization of research of mainly local interest [45,46].

### 4.4. Origin of Published Papers

The results regarding the origin of the published papers reflect the agricultural systems applied on the various countries with regard to the farmed species and also the reliance of these countries on the respective species. The high numbers of sheep farmed in Australia (for wool and meat production), France (primarily for milk production), Italy (primarily for milk production), Spain (for meat and milk production) and the United Kingdom (primarily for meat production) are reflected in the higher number of relevant papers from these countries, as well as in the themes of the relevant papers [47,48]. Moreover, it is also worth mentioning the presence of some emblematic scientific organizations in these countries, among them the Moredun Research Institute and the Rowett Institute (United Kingdom), the Commonwealth Scientific and Industrial Research Organization (Australia), the National Institute for Agricultural Research (rebranded as National Research Institute for Agriculture, Food and Environment) (France), from which important relevant work has originated throughout the study period.

The increased numbers of published papers related to goats from China and India reflect the high numbers of animals in these countries, approximately 130 M and 155 M animals, respectively, which are the highest worldwide [49]. For these two countries, goat farming has a pivotal role in agricultural development [50,51], as discussed above.

The results have indicated a decline in papers related to sheep published by the traditionally leading countries, whilst a significant overall increase was noted in the papers with origin from China. This aligns with the global trend of the significant increase in research output by that country across all scientific disciplines and fields [11]. The increase in published papers related to goats was seen in most countries (although with a steeper slope in papers from China and Brazil), potentially on account of the issues previously discussed.

### 4.5. Research Themes in Published Papers

The central theme in the published papers related to both animal species and throughout the study period was Ruminant nutrition. Nutrition is a primary determinant for animal productivity; it influences growth rate, milk yield and composition, reproductive performance and wool and hair quality [52]. Also, it regulates a plethora of pathways involved in health-related mechanisms, for example, immunological systems (including innate defences), development of metabolic disorders; also, it plays an important role in the welfare of animals [53,54,55,56]. In ruminants, which have a complex digestive tract, nutritional management is of the utmost importance, in order to maintain high productivity and good health of the animals.

Efficient reproductive management is also important for the financial success of sheep and goat farms, in both meat and dairy production systems [57,58,59,60,61,62,63]. Hence, there is a clear need for high reproductive efficiency in farms, which would guarantee a high proportion of animals that lamb or kid (thus ensuring the start of a lactation period) and an increased number of newborn lambs and kids [64,65,66,67]. The above explain the continuous placement of Livestock reproduction as a main theme of the published papers related to both species and throughout the length of the present study.

We hypothesize that the presence of a higher number of topics-micro for published papers related to sheep than for papers on goats, 1760 versus 1573, respectively, possibly reflects the coverage of topics referring to sheep as livestock animals and as biomedical models, as previously discussed [35,36], in contrast to research in goats, which appears to be focused on work within the remit of veterinary and animal sciences, as well as in food production.

### 4.6. Accessibility of Published Papers

The ‘democratization of science’ entails ‘*the public having greater influence over science and that influence being shared more equally among members of the public*’ [68]. As part of this, there has been a significant promotion for the publication of scientific papers under open access.

The ‘open access’ movement started in the early 1990s. Significant milestones in its early years were the Budapest Open Access Initiative (BOAI) and the foundation of the nonprofit organization Creative Commons in the United States, both of which occurred in 2002. Thereafter, the European Union, first among entities of the public sector, supported the movement through the launch of the Open Access in 2008 [69,70,71].

The surge in papers published under open access after 2000 coincides with the above, reflecting the trajectory of the international ‘open science’ approach. Open access publications are particularly valuable for scholars in locations with limited means to access subscription access papers [72], which can be a significant case for research and clinical work on sheep and goats.

### 4.7. Sustainable Development Goals in Published Papers

Classification of papers in accord with sustainable development goals served has been based on topics-micro, which leverage of the usage of the published papers by other authors [73]. Each goal includes a set of topics based on relevant analyses performed on details of the database [73].

We hypothesize that the ‘Good health and well being’ goal was dominant throughout the study period, which may potentially reflect a veterinary and animals science bias in the database. Moreover, we also indicate the clear increase in the proportion of papers that served the ‘Climate action’ goal is more noteworthy. This appears to be in line with the increased output of published papers on Environmental Sciences [29] during the last 10 years. In this context, the sharper increase in this trend among papers related to goats is also noted, which can be aligned to the overall steeper increase in published papers related to goats.

### 4.8. Citations Received by Published Papers

Papers related to sheep have clearly made a more significant impact in research than papers related to goats; the former papers received higher total number of citations, higher average number of citations per paper and higher *h*-index. Papers related to sheep are in general older than papers related to goats; thus, they have had the opportunity to receive more citations. Moreover, this trend can reflect the higher proportion of papers published in English, which allows a wider audience and consequently more citations. Finally, this can be an effect of the increased number of such papers related to topics of biomedical research, which leads to higher citation rates [74,75,76]; these types of papers could have benefited of the significant surge of citations during the period of COVID-19 pandemic [77].

The above are reflected in the differences found in the present results in citation numbers between the top authors for papers related to sheep and papers related to goats: median citations per author 7936 versus 2767, respectively. At the same time, the present results also show a difference in the median time span of papers published by these authors (38 vs. 26 years, respectively) and a difference in the orientation of the research themes (30% vs. 100% within the remit of veterinary and animal science, respectively), which should also be noted.

### 4.9. Authors

The countries of affiliation of the authors with most published papers further corroborate the points discussed above. For papers related to sheep, most of these authors were affiliated with scientific establishments in the United States of America, in full alignment with the country of origin of most such papers. For papers related to goats, most of these authors were affiliated with scientific establishments in China, again in full alignment with a country of origin with many such papers. Moreover, the topics of the published papers by the top authors related to sheep also corroborate that such papers cover topics referring to that species as livestock animals and biomedical models as well.

The top authors with most published papers related to sheep were distinct to those with published papers related to goats. This can be explained by the different nature of work and the differences between the two animal species in management and health problems [15,16,17,18,19,20], which result in different nature of relevant research. Nevertheless, this difference may also be a function of different scientific communities and networks that overlap infrequently.

## 5. Conclusions

The work provided an extensive mapping and analysis of the scientific output related to sheep and goat research internationally. Work on both species shows strong thematic stability. However, changes have been detected in the origin of the published papers in recent years, as well as in the journals used for dissemination of the results. A trend emerges that potentially in the future, new fields of work can develop, for example, climate adaptation research.

## Figures and Tables

**Table 1:** Ten journals, in which most papers related to sheep or goats were published from 1970 to 2024, with respective numbers of published papers.

Papers Related to Sheep			Papers Related to Goats		
Journal in Which Published	*n*	%	Journal in Which Published	*n*	%
*Small Ruminant Research*	3573	2.2	*Small Ruminant Research*	2954	4.4
*Journal of Animal Science*	2520	1.5	*Indian Journal of Animal Sciences*	1628	2.4
*Veterinary Parasitology*	2212	1.3	*Journal of Dairy Science*	1035	1.5
*Animals*	1464	0.9	*Tropical Animal Health and Production*	1011	1.5
*Tropical Animal Health and Production*	1418	0.9	*Animals*	907	1.3
*Plos One*	1394	0.8	*Indian Veterinary Journal*	865	1.3
*Endocrinology*	1392	0.8	*Veterinary Parasitology*	783	1.2
*Indian Journal of Animal Sciences*	1330	0.8	*Theriogenology*	642	0.9
*Biology of Reproduction*	1195	0.7	*Plos One*	619	0.9
*Theriogenology*	1184	0.7	*Veterinary Microbiology*	449	0.7

**Table 2:** Ten Web of Science (WoS) categories of journals, in which most papers related to sheep or goats were published from 1970 to 2024, with respective number of papers.

WoS Categories of Journals					
Papers Related to Sheep			Papers Related to Goats		
Category	*n*	%	Category	*n*	%
Veterinary Sciences	37,235	22.6	Veterinary Sciences	18,554	27.4
Agriculture Dairy Animal Science	27,738	16.1	Agriculture Dairy Animal Science	15,245	22.5
Immunology	9560	5.8	Food Science Technology	5991	8.9%
Biochemistry Molecular Biology	8948	5.4	Biochemistry Molecular Biology	3298	4.9
Parasitology	7158	4.3	Microbiology	2776	4.1
Zoology	7065	4.3	Parasitology	2758	4.1
Reproductive Biology	6963	4.2	Zoology	2754	4.1
Endocrinology Metabolism	6572	4.0	Immunology	2461	3.6
Physiology	6533	4.0	Biotechnology Applied Microbiology	2320	3.4
Agriculture Multidisciplinary	6314	3.8	Reproductive Biology	2132	3.2

**Table 3:** Ten topics-meso of most papers related to sheep or goats published from 1970 to 2024, with respective numbers of published papers.

Topics-Meso			
Papers Related to Sheep	No. of Papers	Papers Related to Goats	No. of Papers
Dairy and Animal Sciences	33,137	Dairy and Animal Sciences	13,436
Parasitology—General	10,635	Food Science and Technology	3385
Immunology	5701	Parasitology—General	3284
Reproductive Biology	5526	Reproductive Biology	2864
Obstetrics and Gynecology	4726	Zoonotic Diseases	2754
Zoonotic Diseases	4126	Virology—General	1457
Zoology and Animal Ecology	3870	Inflammatory Bowel Diseases and Infections	1388
Endocrinology and Metabolism	3502	Zoology and Animal Ecology	1214
Neurodegenerative Diseases	2767	Sexually Transmitted Infections	1187
Bacteriology	2432	Parasitology—Malaria, Toxoplasmosis and Coccidiosis	1127

**Table 4:** Ten topics-micro of most papers related to sheep or goats published from 1970 to 2024, with respective numbers of published papers.

Topics-Micro			
Papers Related to Sheep	No. of Papers	Papers Related to Goats	No. of Papers
Ruminant nutrition	15,323	Ruminant nutrition	5668
Livestock reproduction	8656	Livestock reproduction	3357
Anthelmintic resistance	7130	Dairy product chemistry	2915
Farm animal welfare	3691	Anthelmintic resistance	2278
Wildlife ecology	2822	Farm animal welfare	1233
Prion pathogenesis	2658	Mastitis	1195
Meat quality	2539	Meat quality	1115
Embryo development	2102	Tick-borne pathogens	1082
Tick-borne pathogens	1998	Male fertility	1047
Dairy product chemistry	1673	Wildlife ecology	959

**Table 5:** Top and second top topics-micro of origin on annual basis for papers related to sheep or goats published from 1970 to 2024.

Topics-Micro	Papers Related to Sheep		Papers Related to Goats	
	Years as Top Category	Years as Second Top Category	Years as Top Category	Years as Second Top Category
Anthelmintic resistance	0	6	0	2
Dairy products chemistry	0	0	0	10
Farm animal welfare	0	0	0	1
Interferons	0	2	0	0
Livestock nutrition	0	42	2	39
Mastitis	0	0	0	2
Peroxisomes	0	0	0	1
Ruminant nutrition	55	0	53	0
Schistosomiasis	0	0	0	1
Somatic hypermutation	0	5	1	4

**Table 6:** Details regarding the papers related to sheep or to goats published from 1970 to 2024, published by the ten authors with most of such papers.

	Ten Authors with Most Published Papers	
Attributes	Papers Related to Sheep	Papers Related to Goats
Median no. of papers (IQR ^1^)	214 (56)	133 (23)
Median no. of papers as first author(s) (IQR)	19 (12)	14 (14)
Median no. of papers as last author(s)	101 (41)	45 (29)
Median proportion of collaborative papers among authors with most papers	14.0%	17.4%
Median time span of papers (IQR)	38 (11) years	26 (9) years
Median total citations received by all papers published by each author (IQR)	7936 (5645)	2767 (1183)
Median no. of citations received per paper published by each author (IQR)	24.0 (10.5)	12.0 (3.0)
Median *h*-index (IQR)	50 (16)	26 (8)
Journals in which most papers were published ^2^	*American Journal of Obstetrics and Gynecology*, *American Journal of Physiology-Regulatory Integrative and Comparative Physiology*, *Biology of Reproduction* (*n* = 2 each), *Endocrinology*, *Pediatric Research*, *Shock*, *Small Ruminant Research* (*n* = 1 each)	*Small Ruminant Research* (*n* = 3), *Animals*, *Animal Feed Science and Technology*, *Journal of Dairy Science*, *Molecular Biology Reports*, *Pesquisa Veterinaria Brasileira*, *Theriogenology*, *Veterinary Parasitology* (*n* = 1 each)
Predominant topics-meso ^3^	Obstetrics and gynecology (*n* = 5), Dairy and animal sciences, Wounds and ulcers (*n* = 2 each), Endocrinology and metabolism (*n* = 1)	Dairy and animal sciences (*n* = 5), Reproductive biology (*n* = 3), Complementary and alternative medicine, Parasitology—general (*n* = 1 each)
Predominant topics-micro ^3^	Neonatal hypoxia effects (*n* = 3), Preterm birth causes, Livestock reproduction (*n* = 2), Burns, Gonadotropin-releasing hormone, Mastitis (*n* = 1 each)	Ruminant nutrition (*n* = 5), Fertility preservation (*n* = 3), Anthelmintic resistance, Pyrrolizidine alkaloids (*n* = 1 each)

^1^ IQR: interquartile range; ^2^ results indicate no. of authors with that journal as the predominant in their published papers; ^3^ results indicate no. of authors with that topic as the predominant among in their published papers.

**Figure A1:**
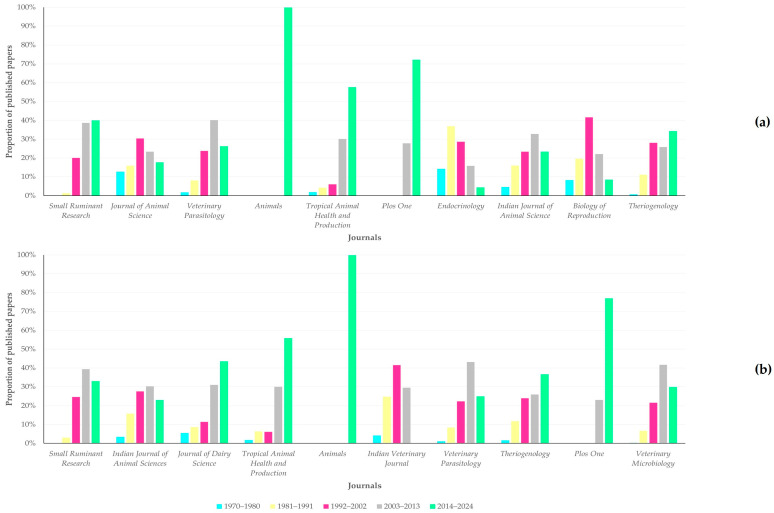
Clustered column plot of the proportion (%) of papers related to sheep (**a**) or to goats (**b**) published from 1970 to 2024 in each of the top ten journals, for respective papers, in accordance with the time period within which publication was done.

**Figure A2:**
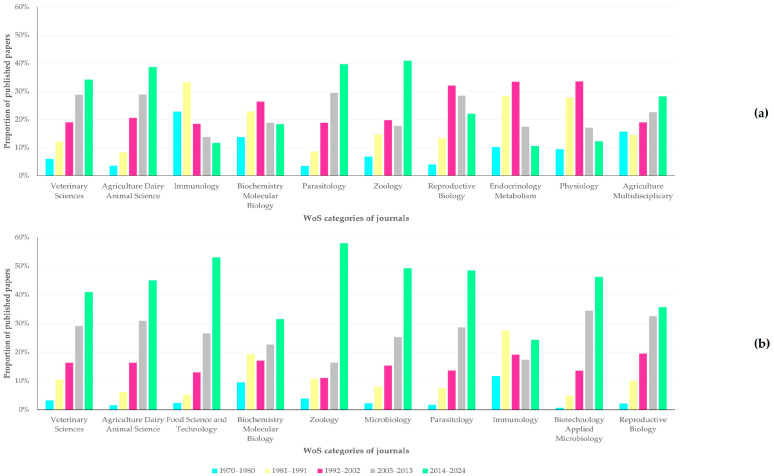
Clustered column plot of the proportion (%) of papers related to sheep (**a**) or to goats (**b**) published from 1970 to 2024 in each of the top ten Web of Science (WoS) categories of journals, for respective papers, in accordance with the time period within which publication was done.

**Figure A3:**
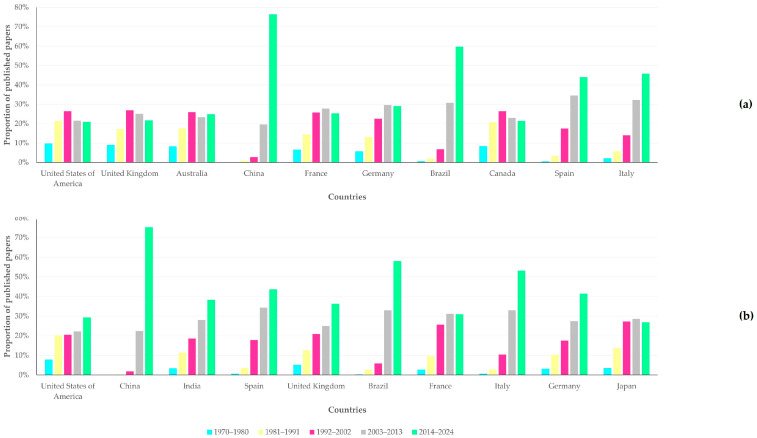
Clustered column plot of the proportion (%) of papers related to sheep (**a**) or to goats (**b**) published from 1970 to 2024 in each of the top ten countries of origin, for respective papers, in accordance with the time period within which publication was done.

**Figure A4:**
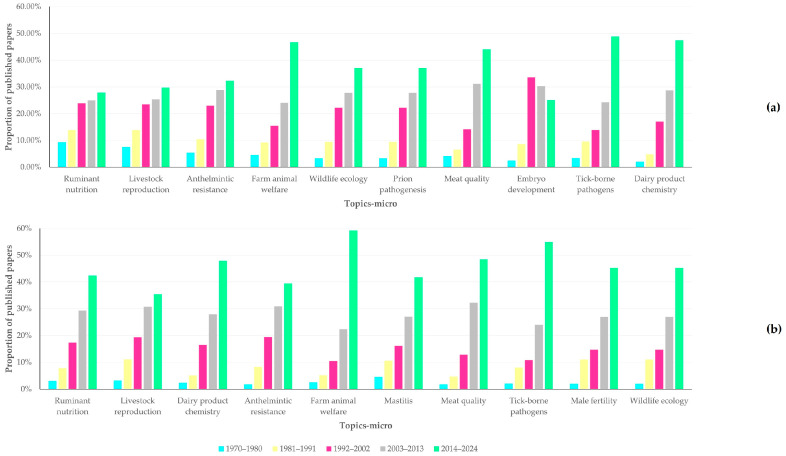
Clustered column plot of the proportion (%) of papers related to sheep (**a**) or to goats (**b**) published from 1970 to 2024 in each of the top ten topics-micro, for respective papers, in accordance with the time period within which publication was done.

## Data Availability

Data related with this study are available in the Supplementary Material.

## Supplementary Materials

The following supporting information can be downloaded at https://www.mdpi.com/article/10.3390/ani16081163/s1, Table S1: Search terms used in TOPIC searches in Web of Science for retrieval of papers published from 1970 to 2024, regarding domestic mammal species. Table S2: Types of documents in Web of Science related to sheep or to goats, published from 1970 to 2024; Table S3: PRISMA flow diagram for the identification and exclusion of records from Web of Science database; Figure S1: Bar plot of the number of papers published from 1970 to 2024 related to sheep or goats in comparison to those of another nine domestic mammal species; Table S4: Proportion of published papers among those related to sheep or goats, in accordance with the time period of publication, and respective slopes during these periods; Table S5: The 100 journals, in which most papers related to sheep or goats were published from 1970 to 2024, with respective numbers of published papers; Figure S2: Bar plot of the proportion of published papers related among the 100 journals, in which most papers related to sheep or to goats were published from 1970 to 2024; Figure S3: ‘Butterfly’-type plot of years of inclusion of journals among the top ten for papers related to sheep or to goats published from 1970 to 2024; Table S6: Number of years, for top and second top journals on annual basis for papers related to sheep or goats published from 1970 to 2024; Table S7: Number of years for journals, in which most papers related to sheep or goats were published annually during the years 1970 to 1980 and 2014 to 2024; Table S8: The 100 Web of Science categories of journals, in which most papers related to sheep or goats were published from 1970 to 2024, with respective numbers of published papers; Figure S4: Bar plot of the proportion of papers related to sheep or to goats and published from 1970 to 2024, in accordance with Web of Science categories of journals; Figure S5: ‘Butterfly’-type plot of years of inclusion of Web of Science categories of journals among the top ten for papers related to sheep or to goats published from 1970 to 2024; Table S9: Number of years for top and second top Web of Science categories of journals on annual basis for papers related to sheep or goats published from 1970 to 2024; Table S10: No. of years for Web of Science categories of journals, in which most papers related to sheep or goats were published annually during the years 1970 to 1980 and 2014 to 2024; Table S11: The languages in which papers related to sheep or goats published from 1970 to 2024 were written, with respective numbers of published papers; Table S12: The 100 countries from which originated most papers related to sheep or goats published from 1970 to 2024, with respective numbers of published papers; Figure S6: Filled maps of countries from which originated at least 1% of papers related to sheep or to goats published from 1970 to 2024, in accordance with respective number of papers; Table S13: Numbers of papers related to sheep or to goats published from 1970 to 2024, in accordance with the geographical subregions of the countries of origin and respective proportions among all relevant papers; Figure S7: ‘Butterfly’-type plot of years of inclusion of countries of origin among the top ten for papers related to sheep or to goats published from 1970 to 2024; Table S14: Number of years for top and second top countries of origin on annual basis for papers related to sheep or goats published from 1970 to 2024; Table S15: Number of years for countries of origin, for most papers related to sheep or goats published annually during the years 1970 to 1980 and 2014 to 2024; Table S16: The 100 scientific organizations with which most papers related to sheep or goats published from 1970 to 2024 were affiliated, with respective numbers of published papers; Table S17: List of countries with the 100 scientific organizations with most papers related to sheep or to goats published from 1970 to 2024, with respective number of scientific organizations; Table S18: The 100 topics-meso of most papers related to sheep or goats published from 1970 to 2024, with respective numbers of published papers; Table S19: The 100 topics-micro of most papers related to sheep or goats published from 1970 to 2024, with respective numbers of published papers; Figure S8: ‘Butterfly’-type plot of years of inclusion of topics-micro among the top ten for papers related to sheep or to goats published from 1970 to 2024; Table S20: Number of years for topics-micro in which most papers related to sheep or goats referred to annually during the years 1970 to 1980 and 2014 to 2024; Figure S9: Yearly proportion of papers related to sheep or goats published from 1970 to 2004 under open-access mode; Table S21: Sustainable development goals served in papers related to sheep or goats published from 1970 to 2024, with respective number of published papers; Figure S10: Column plot of the yearly proportion of papers related to sheep or goats published from 1970 to 2004 in accordance with the Sustainable Development Goals served in the papers; Figure S11: Diagrams of joint authorships among the ten authors with most papers on sheep (a) or goats (b) published from 1970 to 2024 (letters within the circles denote authors, but do not correspond to their surnames in order to guarantee private data protection).
