# Supplementary material for: Mapping the Scientific Literature on Sheep and Goat Research: General Appraisal and Significance of the Year of Publication
Source: Animals (Basel). 2026 Apr 10;16(8):1163. doi: 10.3390/ani16081163 (PMC13113982; doi:10.3390/ani16081163)

Mapping the Scientific Literature on Sheep and Goat Research: General Appraisal and Significance of the Year of Publication

Table S1. Search terms used in searches in Web of Science for retrieval of papers published from 1970 to 2024, regarding domestic mammal species.

TOPIC	AND	YEAR PUBLISHED
[buffalo* OR bubaline OR *Bubalus bubalis*]	AND	[1970-2024]
[camel* OR *Camelus bactrianus* OR *Camelus dromedarius*]	AND	[1970-2024]
[cat* OR feline OR *Felis catus*]	AND	[1970-2024]
[cattle OR bovine or *Bos taurus*]	AND	[1970-2024]
[dog* OR canine OR *Canis familiaris*]	AND	[1970-2024]
[donkey* OR asinine OR *Equus asinus*]	AND	[1970-2024]
[goat* OR caprine OR *Capra hircus*]	AND	[1970-2024]
[horse* OR equine OR *Equus caballus*]	AND	[1970-2024]
[pig* OR swine OR *Sus scrofa*]	AND	[1970-2024]
[rabbit* OR lapine OR *Oryctolagus cuniculus*]	AND	[1970-2024]
[sheep OR ovine OR *Ovis aries*]	AND	[1970-2024]

Table S2. Types of documents (in alphabetical order) in Web of Science related to sheep or to goats, published from 1970 to 2024, with respective numbers (*n*).

Types of documents related to sheep	*n*	Types of documents related to goats	*n*
Abstract of Published Item	3	Abstract of Published Item	2
Art exhibit review	7	Art exhibit review	7
Article	159,518	Article	65,104
Bibliography	2	Bibliography	1
Biographical-item	17	Biographical-item	3
Book chapters	128	Book chapters	56
Book review	352	Book review	217
Correction	348	Correction	168
Correction, addition	46	Correction, addition	9
Data paper	31	Data paper	13
Discussion	15	Discussion	3
Early access	100	Early access	55
Editorial material	1330	Editorial material	411
Excerpt	2	Excerpt	1
Fiction, creative prose	15	Fiction, creative prose	18
Film review	18	Film review	4
Hardware review	1	Item About an Individual	2
Item About an Individual	5	Letter	405
Letter	1246	Meeting	2
Meeting	4	Meeting abstract	3328
Meeting abstract	12,178	Music performance review	2
Music performance review	3	News item	94
Music score review	2	Note	1875
News item	364	Poetry	74
Note	3976	Proceeding paper	1650
Poetry	98	Publication with expression of concern	1
Proceeding paper	4751	Record review	12
Publication with expression of concern	2	Reprint	5
Record review	4	Retracted publication	17
Reprint	35	Retraction	4
Retracted publication	44	Review article	2533
Retraction	8	Theater review	9
Review article	5532	TV review, radio review video	1
Script	1		
Theater review	10		
TV review, radio review video	2		

Table S3. PRISMA flow diagram for the identification and exclusion of records from Web of Science database.

(a) Papers related to sheep

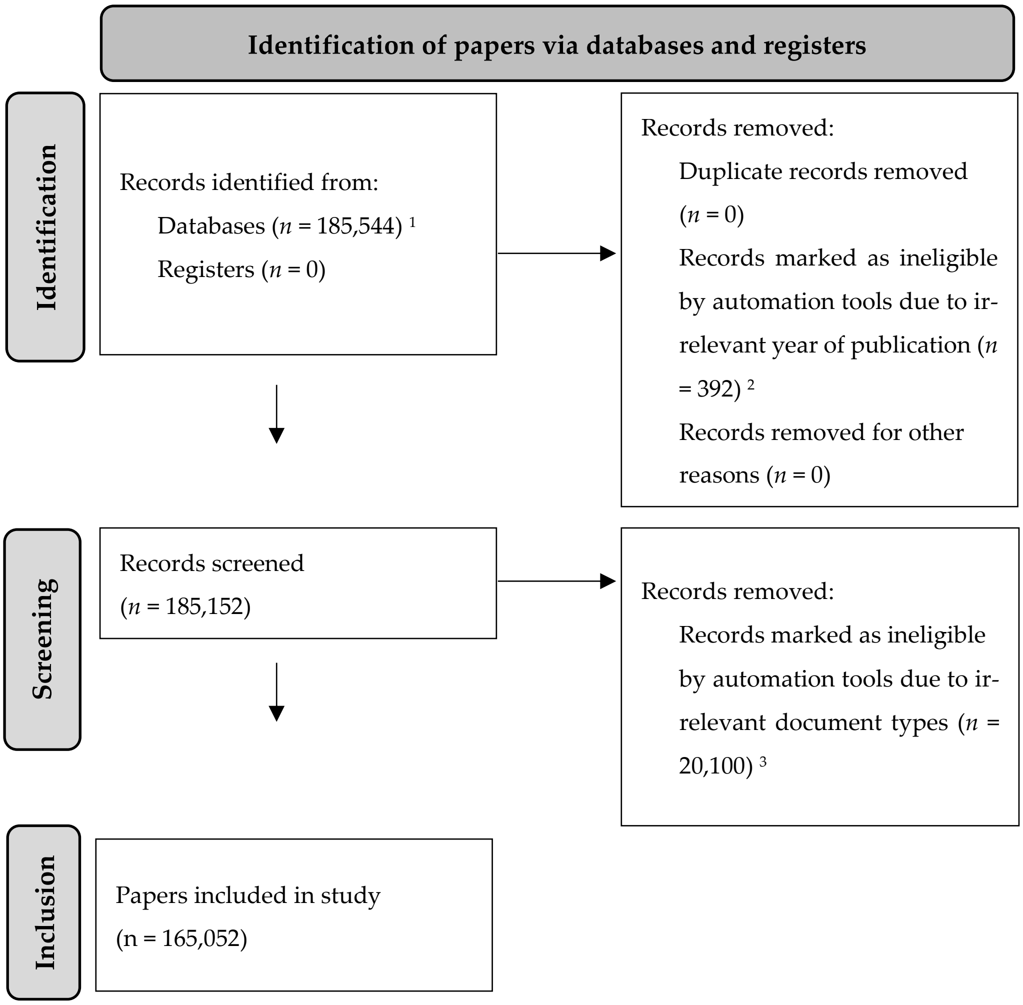

(b) Papers related to goats

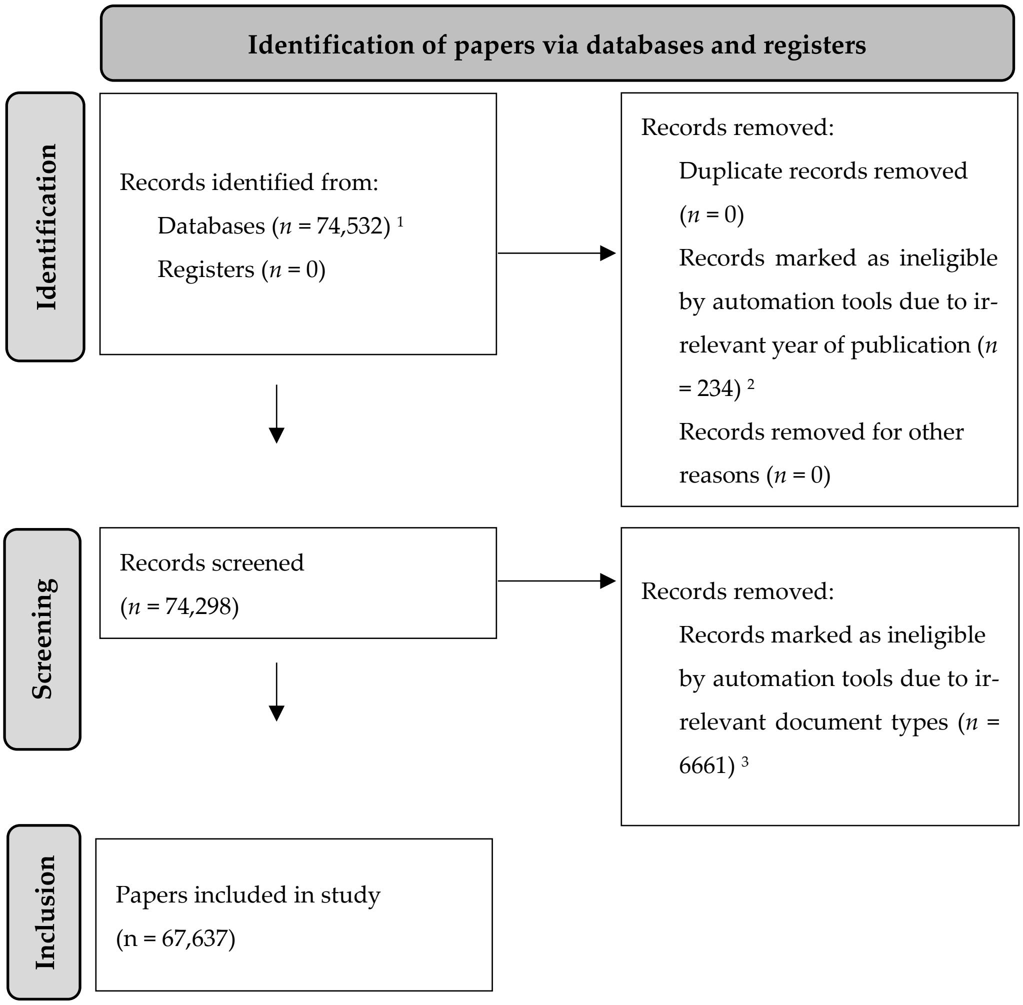

[1] Record search performed in Web of Science database; [2] Records with [Publication year] or [Final publication year] 2025 or 2026 excluded – exclusion performed by the Web of Science platform; [3] Papers excluded for not being ‘Articles’ or Review articles’ – exclusion performed by the Web of Science platform (details in Table S3).

Figure S1. Bar plot of the number of papers (n) published from 1970 to 2024 related to sheep (green) or goats (coral) in comparison to those of another nine domestic mammal species.

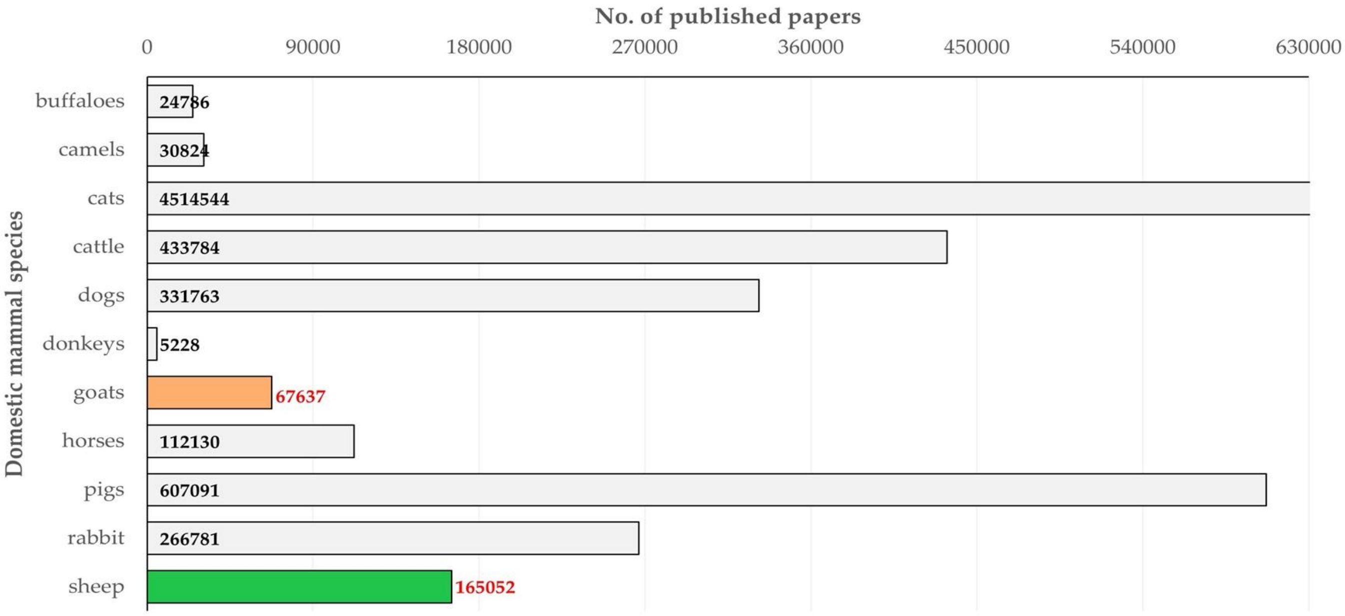

Table S4. Proportion of published papers among those related to sheep or goats, in accordance with the time period of publication (from 1970 to 2024), and respective slopes during these periods.

**Time period (no. of years)**	**Proportion of Published Papers Among All Papers**	
	**Related to Sheep**	**Related to Goats**
1970-1980 (*n* = 11)	8.0%<br>0.0011 ± 0.0002	3.9%<br>0.0007 ± 0.0000
1981-1991 (*n* = 11)	15.0%<br>0.0003 ± 0.0002	10.3%<br>0.0003 ± 0.0000
1992-2002 (*n* = 11)	21.6%<br>0.0001 ± 0.0000	16.1%<br>0.0005 ± 0.0000
2003-2013 (*n* = 11)	24.9%<br>0.0007 ± 0.0000	27.2%<br>0.0016 ± 0.0001
2014-2024 (*n* = 11)	30.4%<br>0.0010 ± 0.0002	42.5%<br>0.0023 ± 0.0003

Table S5. The 100 journals (in order of results count), in which most papers related to sheep or goats were published from 1970 to 2024, with respective numbers (*n*) of published papers.

Papers Related to Sheep		Papers Related to Goats	
**Journals**	***n***	**Journals**	***n***
Small Ruminant Research	3573	*Small Ruminant Research*	2954
Journal of Animal Science	2520	*Indian Journal of Animal Sciences*	1628
Veterinary Parasitology	2212	*Journal of Dairy Science*	1035
Animals	1464	*Tropical Animal Health and Production*	1011
Tropical Animal Health and Production	1418	*Animals*	907
Plos One	1394	*Indian Veterinary Journal*	865
Endocrinology	1392	*Veterinary Parasitology*	783
Indian Journal of Animal Sciences	1330	*Theriogenology*	642
Biology of Reproduction	1195	*Plos One*	619
Theriogenology	1184	*Veterinary Microbiology*	449
Research in Veterinary Science	1139	*Asian Australasian Journal of Animal Sciences*	438
Veterinary Record	1125	*Journal of Animal Science*	414
Animal Feed Science and Technology	1105	*Research in Veterinary Science*	400
Journal of Dairy Science	1079	*Animal Reproduction Science*	392
Veterinary Microbiology	1009	*Frontiers in Veterinary Science*	391
Journal of Immunology	1004	*Journal of Dairy Research*	350
Journal of Endocrinology	911	*Indian Journal of Animal Research*	348
Journal of Applied Physiology	905	*Veterinary Record*	334
Journal of Agricultural Science	887	*Pesquisa Veterinaria Brasileira*	327
Animal Reproduction Science	879	*Scientific Reports*	316
Animal Production Science	811	*International Dairy Journal*	315
Australian Journal of Agricultural Research	770	*BMC Veterinary Research*	308
Australian Veterinary Journal	766	*American Journal of Veterinary Research*	304
American Journal of Veterinary Research	751	*Animal Feed Science and Technology*	299
Journal of Reproduction and Fertility	735	*Journal of Applied Animal Research*	294
American Journal of Physiology	733	*Food Chemistry*	292
American Journal of Obstetrics and Gynecology	723	*Reproduction on Domestic Animals*	282
Animal	705	*Journal of Agricultural and Food Chemistry*	270
Veterinary Immunology and Immunopathology	692	*Revista Brasileira de Zootecnia*	268
Indian Veterinary Journal	666	*Journal of Immunology*	264
Scientific Reports	660	*Parasitology Research*	256
Asian Australasian Journal of Animal Sciences	623	*Livestock Science*	253
Parasitology Research	617	*Animal*	252
Pediatric Research	616	*Preventive Veterinary Medicine*	239
British Journal of Nutrition	613	*Transboundary and Emerging Diseases*	238
Australian Journal of Experimental Agriculture	610	*Journal of Animal and Veterinary Advances*	237
Livestock Science	609	*Journal of Immunological Methods*	233
Applied Animal Behaviour Science	606	*Turkish Journal of Veterinary Animal Sciences*	228
Immunology	602	*Veterinary Research Communications*	228
Journal of Physiology London	601	*Arquivo Brasileiro de Medicina Veterinaria e Zootecnia*	226
Frontiers in Veterinary Science	575	*Foods*	226
Pesquisa Veterinaria Brasileira	563	*Italian Journal of Animal Science*	219
International Journal for Parasitology	562	*Animal Biotechnology*	207
Journal of Biological Chemistry	550	*Revue de Medecine Veterinaire*	206
Journal of Comparative Pathology	545	*Applied Animal Behaviour Science*	202
Meat Science	543	*Journal of Veterinary Diagnostic Investigation*	193
Revista Brasileira de Zootecnia	540	*South African Journal of Animal Science*	193
New Zealand Journal of Agricultural Research	525	*Meat Science*	190
Preventive Veterinary Medicine	518	*Acta Tropica*	180
Canadian Journal of Animal Science	505	*Journal of Comparative Pathology*	180
American Journal of Physiology Regulatory Integrative and Comparative Physiology	492	*Journal of the American Veterinary Medical Association*	180
New Zealand Veterinary Journal	489	*Animal Production Science*	179
Reproduction Fertility and Development	476	*Veterinary Research*	177

Reproduction in Domestic Animals	472	*Journal of Veterinary Pharmacology and Therapeutics*	176
Infection and Immunity	467	*Animal Science Journal*	173
BMC Veterinary Research	463	*Pakistan Journal of Zoology*	173
Cellular Immunology	448	*Veterinary Immunology and Immunopathology*	173
Turkish Journal of Veterinary Animal Sciences	445	*Journal of Applied Physiology*	169
Journal of Clinical Microbiology	437	*Onderstepoort Journal of Veterinary Research*	167
Animal Genetics	412	*Milchwissenschaft*	163
Animal Science	411	*Journal of Clinical Microbiology*	162
Journal of Immunological Methods	410	*Comparative Immunology Microbiology and Infectious Diseases*	159
Revue de Medecine Veterinaire	408	*Frontiers in Microbiology*	157
Veterinary Research Communications	391	*Parasites Vectors*	155
Journal of Virology	382	*Viruses Basel*	152
Arquivo Brasileiro de Medicina Veterinaria e Zootecnia	380	*International Journal of Molecular Sciences*	150
Veterinary Research	377	*International Journal of Dairy Technology*	148
Biochemical Journal	376	*Journal of Veterinary Medical Science*	148
Parasitology	376	*Pathogens*	148
Semina Ciencias Agrarias	374	*Journal of Virological Methods*	147
Animal Production	367	*Kafkas Universitesi Veteriner Fakultesi Dergisi*	145
Journal of Veterinary Diagnostic Investigation	365	*Pakistan Veterinary Journal*	144
General and Comparative Endocrinology	361	*Journal of Agricultural Science*	143
Domestic Animal Endocrinology	360	*Animal Genetics*	141
Journal of General Virology	359	*Journal of Animal Physiology and Animal Nutrition*	140
Livestock Production Science	358	*Semina Ciencias Agrarias*	140
Biochemical and Biophysical Research Communications	356	*Lwt Food Science and Technology*	139
Journal of Animal and Veterinary Advances	354	*Journal of Animal and Plant Sciences*	132
Grass and Forage Science	351	*Acta Veterinaria Scandinavica*	131
Vaccine	351	*Journal of Virology*	131
Onderstepoort Journal of Veterinary Research	346	*Gene*	130
South African Journal of Animal Science	344	*Veterinary Journal*	130
Italian Journal of Animal Science	338	*Revue Scientifique et Technique Office International des Epizooties*	129
Proceedings of The National Academy of Sciences of The United States of America	337	*Veterinary Sciences*	125
Journal of Veterinary Pharmacology and Therapeutics	334	*Journal of Food Protection*	122
Molecular and Cellular Endocrinology	332	*Vaccine*	122
Clinical and Experimental Immunology	327	*Journal of Arid Environments*	120
Reproduction	321	*Veterinary Medicine and Science*	120
Parasites Vectors	320	*BMC Genomics*	119
Biochimica et Biophysica Acta	314	*Molecular Biology Reports*	118
Indian Journal of Animal Research	314	*Acta Scientiae Veterinariae*	117
Journal of Applied Animal Research	314	*Australian Veterinary Journal*	117
Journal of Wildlife Diseases	313	*Journal of Endocrinology*	115
Transboundary and Emerging Diseases	311	*Journal of Biological Chemistry*	114
Journal of Animal Physiology and Animal Nutrition	296	*Journal of the Science of Food and Agriculture*	113
Journal of the Science of Food and Agriculture	295	*Artificial Organs*	112
Applied and Environmental Microbiology	293	*Plos Neglected Tropical Diseases*	111
Veterinary Journal	292	*Vector Borne and Zoonotic Diseases*	110
Acta Veterinaria Scandinavica	290	*Veterinary Pathology*	110
Journal of Developmental Physiology	288	*Journal of General Virology*	109

Figure S2. Bar plot of the proportion (%) of published papers related among the 100 journals (in alphabetical order), in which most papers related to sheep (green) or to goats (coral) were published from 1970 to 2024.

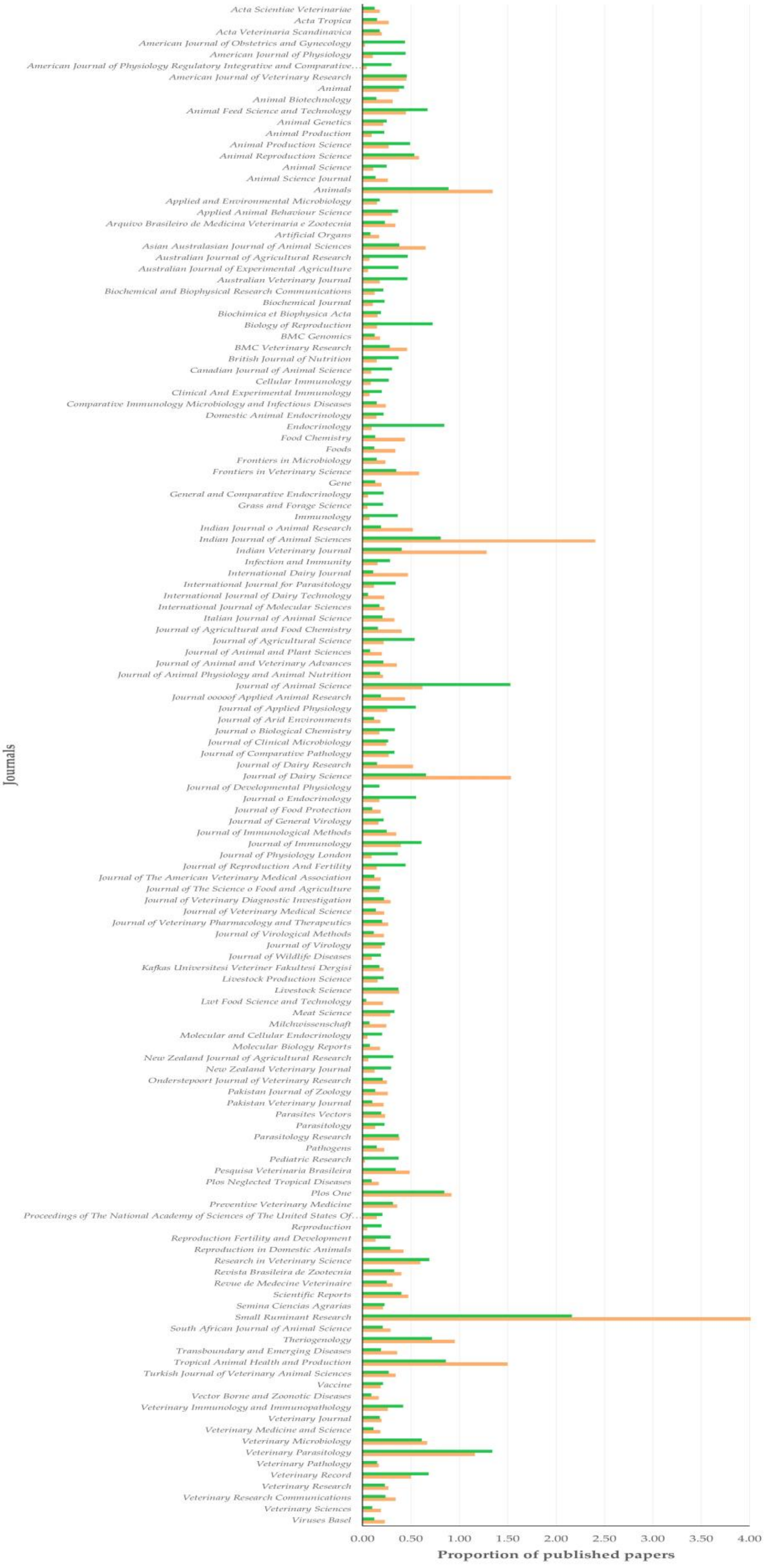

Figure S3. 'Butterfly'-type plot of years [1] (n) of inclusion of journals among the top ten for papers related to sheep (green) or to goats (coral) published from 1970 to 2024 (year at edge of bar indicates median year of publication of respective published papers [2]).

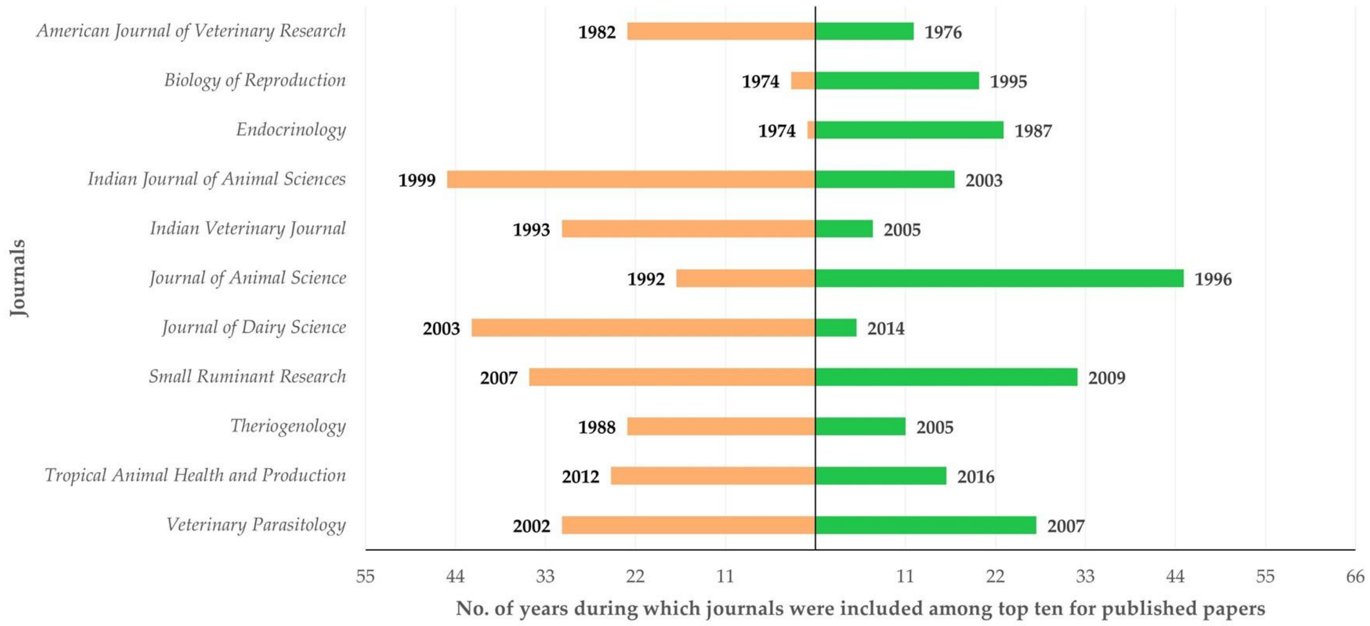

[1] Length of bars indicates the number of years during which journals were included among the top ten categories for published papers.

[2] Number at edge of bars indicates the median year of publication of respective published papers during these years.

Table S6. Number of years (*n*), for top and second top journals on annual basis for papers related to sheep or goats published from 1970 to 2024.

Journals	Papers Related to Sheep		Papers Related to Goats	
	Years as top journal (*n*)	Years as second top journal (*n*)	Years as top journal (*n*)	Years as second top journal (*n*)
Acta Veterinaria Scandinavica	0	0	0	1
American Journal of Physiology	1	5	0	0
American Journal of Veterinary Research	0	0	3	5
Animal Production Science	0	1	0	0
Animals	5	1	5	1
Annales de Biologie Animale Biochimie Biophysique	0	0	0	1
Asian Australasian Journal of Animal Sciences	1	0	0	0
Australian Journal of Agricultural Research	0	4	0	0
Australian Veterinary Journal	0	1	0	0
Biochimica et Biophysica Acta	0	0	0	1
Canadian Journal of Animal Science	1	0	0	0
Cellular Immunology	0	2	0	0
Endocrinology	5	4	0	0
Frontiers in Veterinary Science	0	3	0	3
Immunology	0	2	0	0
Indian Journal of Animal Research	0	0	0	1
Indian Journal of Animal Sciences	0	0	10	12
Indian Journal of Experimental Biology	0	0	0	14
Indian Veterinary Journal	0	0	3	0
Journal of Agricultural Science	2	1	0	0
Journal of Animal and Veterinary Advances	0	0	0	1
Journal of Animal Science	11	7	0	0
Journal of Applied Physiology	1	1	0	0
Journal of Comparative Pathology	0	0	0	1
Journal of Dairy Research	0	0	1	0
Journal of Dairy Science	0	0	2	0
Journal of Endocrinology	0	0	1	1
Journal of Helminthology	0	0	0	1
Journal of Immunological Methods	0	0	0	1
Journal of Immunology	8	2	2	2
Journal of Physiology London	0	0	0	1
Pathologia Veterinaria	0	0	0	1
Plos One	1	5	0	5
Research on Veterinary Science	1	0	0	0
Small Ruminant Research	21	7	29	2
South African Journal of Animal Science	0	0	0	1
Tropical Animal Health and Production	0	0	0	4
Veterinary Parasitology	0	11	0	0

Table S7. Number of years (*n*) for journals (in alphabetical order), in which most papers related to sheep or goats were published annually during the years 1970 to 1980 and 2014 to 2024.

Papers Related to Sheep			
**Period 1970-1980**		**Period 2014-2024**	
Journals	no. of years	Journals	no. of years
American Journal of Physiology	1	*Animal*	3
American Journal of Veterinary Research	7	*Animal Biotechnology*	1
Animal Production	2	*Animal Production Science*	9
Annales de Recherches Veterinaires	1	*Animals*	6
Australian Journal of Agricultural Research	7	*BMC Veterinary Research*	3
Australian Journal of Biological Sciences	2	*Foods*	1
Australian Journal of Experimental Agriculture	1	*Frontiers in Veterinary Science*	5
Australian Journal of Experimental Biology and Medical Science	2	*Indian Journal of Animal Research*	1
Australian Veterinary Journal	4	*Indian Journal of Animal Sciences*	3
Biochimica et Biophysica Acta	2	*International Journal of Molecular Sciences*	2
Biology of Reproduction	2	*Journal of Animal Science*	5
British Journal of Nutrition	7	*Journal of Dairy Science*	3
Cellular Immunology	5	*Livestock Science*	2
Clinical and Experimental Immunology	4	*Meat Science*	1
Clinical Immunology and Immunopathology	1	*Pathogens*	2
Comptes Rendus Hebdomadaires des Seances de l' Academie des Sciences Serie D	1	*Pesquisa Veterinaria Brasileira*	2
Cornell Veterinarian	1	*Plos One*	11
Endocrinology	5	*Scientific Reports*	8
General and Comparative Endocrinology	1	*Semina Ciencias Agrarias*	2
Immunology	5	*Small Ruminant Research*	11
Infection and Immunity	2	*Theriogenology*	4
Journal of Agricultural Science	10	*Transboundary and Emerging Diseases*	1
Journal of Animal Science	11	*Tropical Animal Health and Production*	11
Journal of Comparative Pathology	3	*Veterinary Medicine and Science*	1
Journal of Endocrinology	3	*Veterinary Microbiology*	1
Journal of Experimental Medicine	1	*Veterinary Parasitology*	7
Journal of Immunological Methods	2	*Veterinary Sciences*	1
Journal of Immunology	6	*Viruses Basel*	1
Journal of Reproduction And Fertility	2		
New Zealand Journal of Agricultural Research	2		
New Zealand Journal of Agriculture	2		
Proceedings of the Society for Experimental Biology and Medicine	1		
Research in Veterinary Science	6		
Veterinary Record	5		
**Papers Related to Goats**			
**Period 1970-1980**		**Period 2014-2024**	
Journals	no. of years	Journals	no. of years
Acta Neuropathologica	1	*Animal Biotechnology*	1
Acta Physiologica Scandinavica	4	*Animal Science Journal*	1
Acta Veterinaria Academiae Scientiarum Hungaricae	1	*Animals*	6
Acta Veterinaria Scandinavica	3	*Asian Australasian Journal of Animal Sciences*	2
American Journal of Physiology	3	*BMC Veterinary Research*	4
American Journal of Veterinary Research	10	*Foods*	5
Analytical Biochemistry	1	*Frontiers in Microbiology*	1
Anatomia Histologia Embryologia	1	*Frontiers in Veterinary Science*	5
Anatomischer Anzeiger	1	*Genetics and Molecular Research*	2
Animal Production	1	*Indian Journal of Animal Research*	5
Annales de Biologie Animale Biochimie Biophysique	3	*Indian Journal of Animal Sciences*	8
Annales de Recherches Veterinaires	1	*International Dairy Journal*	1
Annales de Zootechnie	1	*International Journal of Molecular Sciences*	1

Annals of The New York Academy of Sciences	1	*Journal of Applied Animal Research*	1
Archives Internationales de Pharmacodynamie et de Therapie	2	*Journal of Dairy Science*	11
Archives of Biochemistry and Biophysics	1	*Mitochondrial DNA Part A*	1
Australian Journal of Experimental Biology and Medical Science	1	*Pathogens*	1
Biochemical Journal	1	*Pesquisa Veterinaria Brasileira*	1
Biochemistry	1	*Plos One*	8
Biochemical and Biophysical Research Communications	1	*Reproduction in Domestic Animals*	1
Biochimica et Biophysica Acta	7	*Scientific Reports*	8
Biology of Reproduction	3	*Small Ruminant Research*	11
Boletin de la Oficina Sanitaria Panamericana	1	*Theriogenology*	4
British Veterinary Journal	5	*Transboundary and Emerging Diseases*	4
Canadian Journal of Comparative Medicine	1	*Tropical Animal Health And Production*	11
Canadian Journal of Microbiology	1	*Veterinary Microbiology*	2
Cellular Immunology	3	*Veterinary Parasitology*	4
Clinical and Experimental Immunology	2	*Veterinary Sciences*	1
Comparative Biochemistry and Physiology B Biochemistry Molecular Biology	1	*Veterinary World*	2
Comptes Rendus Hebdomadaires des Seances de l' Academie des Sciences Serie D	2	*Viruses Basel*	1
Cornell Veterinarian	1		
Endocrinology	1		
Experientia	1		
Indian Journal of Animal Sciences	5		
Indian Journal of Biochemistry Biophysics	2		
Indian Journal of Experimental Biology	2		
Indian Journal of Medical Research	2		
Indian Veterinary Journal	3		
Infection and Immunity	3		
International Journal of Biochemistry	1		
International Journal of Radiation Biology and Related Studies in Physics Chemistry and Medicine	1		
Japanese Journal of Veterinary Science	2		
Journal of Agricultural and Food Chemistry	2		
Journal of Agricultural Science	1		
Journal of Animal Science	4		
Journal of Applied Physiology	2		
Journal of Biological Chemistry	2		
Journal of Comparative Pathology	3		
Journal of Dairy Research	7		
Journal Of Dairy Science	1		
Journal of Endocrinology	5		
Journal of Experimental Medicine	1		
Journal of General Physiology	1		
Journal of Helminthology	1		
Journal of Immunological Methods	4		
Journal of Immunology	6		
Journal of Infectious Diseases	1		
Journal of Physiology London	2		
Journal of Range Management	1		
Journal of Reproduction and Fertility	1		
Journal of the American Veterinary Medical Association	2		
Journal of the National Cancer Institute	1		
Journal of Wildlife Management	1		
Journal of Zoology	1		
Nature	2		

New Zealand Journal of Agriculture	1
New Zealand Journal of Science	1
Nordisk Veterinaer Medicin	3
Pathologia Veterinaria	1
Proceedings of the National Academy of Sciences of The United States of America	3
Proceedings of the Society for Experimental Biology and Medicine	2
Prostaglandins	1
Research in Veterinary Science	7
Respiration Physiology	1
Schweizerische Landwirtschaftliche Monatshefte	1
Texas Agricultural Experiment Station Progress Report	1
Tropical Animal Health and Production	2
Veterinary Medicine and Small Animal Clinician	1
Veterinary Parasitology	1
Veterinary Record	5
Zeitschrift fur Zellforschung und Mikroskopische Anatomie	3
Zentralblatt fur Veterinarmedizin Reihe A	4
Zentralblatt Fur Veterinarmedizin Reihe B	1

Table S8. The 100 Web of Science categories of journals (in order of results count), in which most papers related to sheep or goats were published from 1970 to 2024, with respective numbers (*n*) of published papers.

Papers Related to Sheep		Papers Related to Goats	
**Category of journals**	***n***	**Category of journals**	***n***
Veterinary Sciences	37,235	Veterinary Sciences	18,579
Agriculture Dairy Animal Science	27,738	Agriculture Dairy Animal Science	15,257
Immunology	9560	Food Science Technology	6036
Biochemistry Molecular Biology	8948	Biochemistry Molecular Biology	3320
Parasitology	7158	Zoology	2832
Zoology	7065	Microbiology	2793
Reproductive Biology	6963	Parasitology	2776
Endocrinology Metabolism	6572	Immunology	2472
Physiology	6533	Biotechnology Applied Microbiology	2347
Agriculture Multidisciplinary	6314	Reproductive Biology	2133
Microbiology	6224	Agriculture Multidisciplinary	2109
Food Science Technology	5767	Multidisciplinary Sciences	1679
Pharmacology Pharmacy	4879	Ecology	1552
Cell Biology	4509	Genetics Heredity	1541
Ecology	4497	Infectious Diseases	1483
Biotechnology Applied Microbiology	4095	Environmental Sciences	1457
Multidisciplinary Sciences	3889	Cell Biology	1443
Genetics Heredity	3594	Pharmacology Pharmacy	1253
Environmental Sciences	3526	Chemistry Applied	1215
Neurosciences	3375	Physiology	1204
Surgery	3312	Biology	1182
Medicine Research Experimental	3126	Virology	1050
Infectious Diseases	2814	Agronomy	1034
Cardiac Cardiovascular Systems	2804	Biochemical Research Methods	1016
Engineering Biomedical	2620	Chemistry Analytical	1014
Agronomy	2561	Endocrinology Metabolism	910
Biology	2478	Nutrition Dietetics	874
Obstetrics Gynecology	2391	Tropical Medicine	874
Developmental Biology	2309	Public Environmental Occupational Health	846
Virology	2154	Medicine Research Experimental	842
Respiratory System	1938	Engineering Biomedical	826
Toxicology	1938	Plant Sciences	704
Biophysics	1935	Toxicology	684
Nutrition Dietetics	1825	Biophysics	680
Pathology	1789	Chemistry Multidisciplinary	626
Orthopedics	1732	Pathology	581
Public Environmental Occupational Health	1719	Orthopedics	567
Biochemical Research Methods	1690	Surgery	553
Pediatrics	1575	Archaeology	549
Behavioral Sciences	1422	Developmental Biology	514
Sport Sciences	1381	Neurosciences	499
Peripheral Vascular Disease	1345	Anatomy Morphology	478
Tropical Medicine	1344	Behavioral Sciences	456
Plant Sciences	1340	Biodiversity Conservation	456
Chemistry Applied	1279	Materials Science Biomaterials	454
Materials Science Biomaterials	1260	Geosciences Multidisciplinary	382
Hematology	1245	Cardiac Cardiovascular Systems	379
Biodiversity Conservation	1218	Anthropology	350
Chemistry Analytical	1166	Medicine General Internal	331
Chemistry Multidisciplinary	1097	Entomology	329
Medicine General Internal	1095	Oncology	317
Oncology	1029	Sport Sciences	306
Anatomy Morphology	988	Transplantation	281
Clinical Neurology	967	Clinical Neurology	256

Critical Care Medicine	951	Respiratory System	255
Entomology	906	Evolutionary Biology	249
Archaeology	834	Materials Science Multidisciplinary	248
Soil Science	787	Medical Laboratory Technology	248
Radiology Nuclear Medicine Medical Imaging	772	Forestry	240
Geosciences Multidisciplinary	750	Marine Freshwater Biology	228
Evolutionary Biology	725	Environmental Studies	226
Dentistry Oral Surgery Medicine	670	Hematology	224
Transplantation	657	Materials Science Textiles	210
Chemistry Medicinal	614	Green Sustainable Science Technology	205
Anthropology	578	Nanoscience Nanotechnology	204
Anesthesiology	570	Engineering Chemical	196
Cell Tissue Engineering	475	Obstetrics Gynecology	189
Environmental Studies	431	Peripheral Vascular Disease	184
Urology Nephrology	430	Chemistry Physical	182
Forestry	420	Polymer Science	182
Materials Science Multidisciplinary	358	Chemistry Medicinal	180
Fisheries	352	Physics Applied	173
Medical Laboratory Technology	350	Dentistry Oral Surgery Medicine	171
Dermatology	346	Soil Science	167
Engineering Environmental	338	Cell Tissue Engineering	158
Green Sustainable Science Technology	333	Fisheries	153
Allergy	329	Geography Physical	148
Geography Physical	314	Pediatrics	139
Psychology Biological	288	Engineering Environmental	137
Ophthalmology	279	Instruments Instrumentation	134
Otorhinolaryngology	270	Electrochemistry	126
Engineering Chemical	254	Spectroscopy	126
Marine Freshwater Biology	252	Ophthalmology	121
Water Resources	252	Energy Fuels	118
Materials Science Textiles	248	Allergy	113
Gastroenterology Hepatology	243	Radiology Nuclear Medicine Medical Imaging	110
Polymer Science	238	Dermatology	109
Rheumatology	230	Engineering Electrical Electronic	102
Meteorology Atmospheric Sciences	225	Agricultural Engineering	92
History	222	Meteorology Atmospheric Sciences	86
Emergency Medicine	221	Agricultural Economics Policy	85
Physics Applied	212	Psychology Biological	84
Chemistry Physical	211	Gastroenterology Hepatology	82
Computer Science Interdisciplinary Applications	206	Urology Nephrology	81
Energy Fuels	201	Rheumatology	80
Integrative Complementary Medicine	201	Water Resources	80
Acoustics	196	Engineering Multidisciplinary	78
Agricultural Economics Policy	185	Economics	76
Economics	181	Oceanography	76
Mathematical Computational Biology	180	Microscopy	75

Figure S4. Bar plot of the proportion (%) of papers related to sheep (green) or to goats (coral) and published from 1970 to 2024, in accordance with Web of Science (WoS) categories of journals.

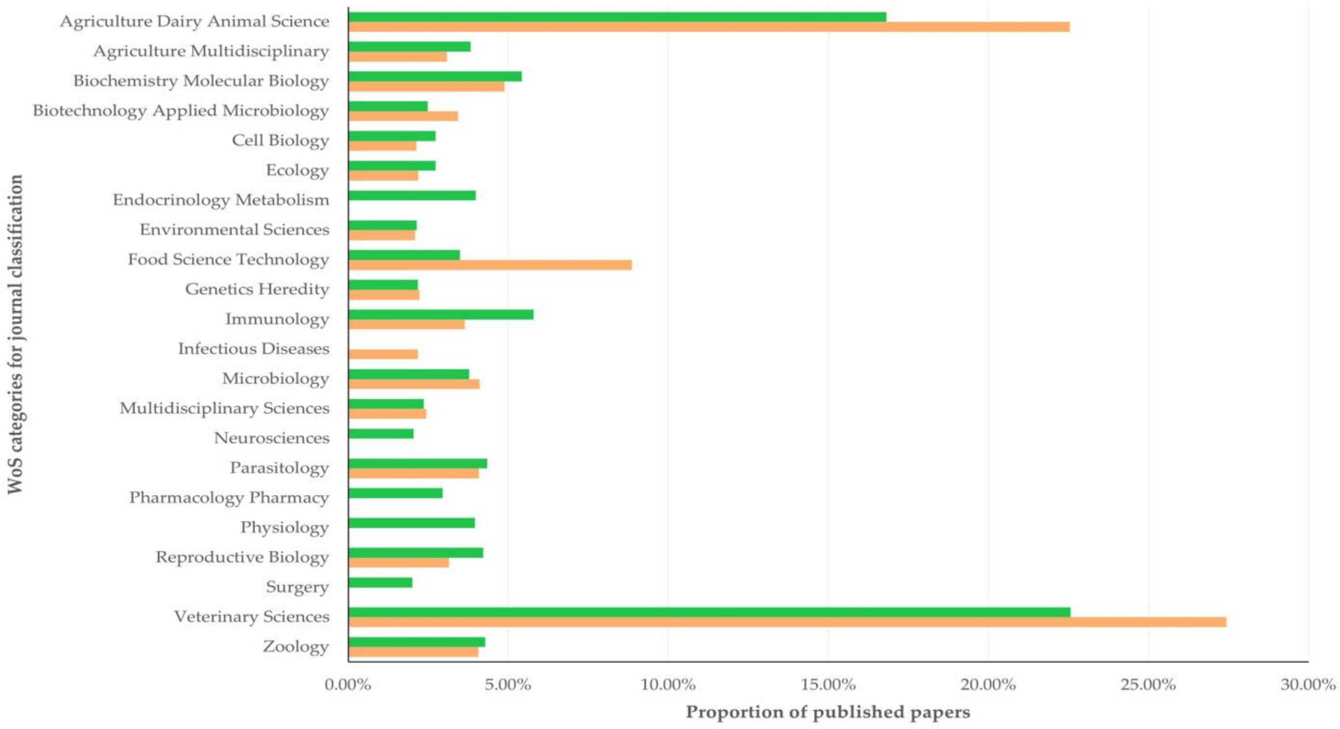

Figure S5. ‘Butterfly’-type plot of years [1] (n) of inclusion of Web of Science categories of journals among the top ten for papers related to sheep (green) or to goats (coral) published from 1970 to 2024 (year at edge of bar indicates median year of publication of respective published papers [2]).

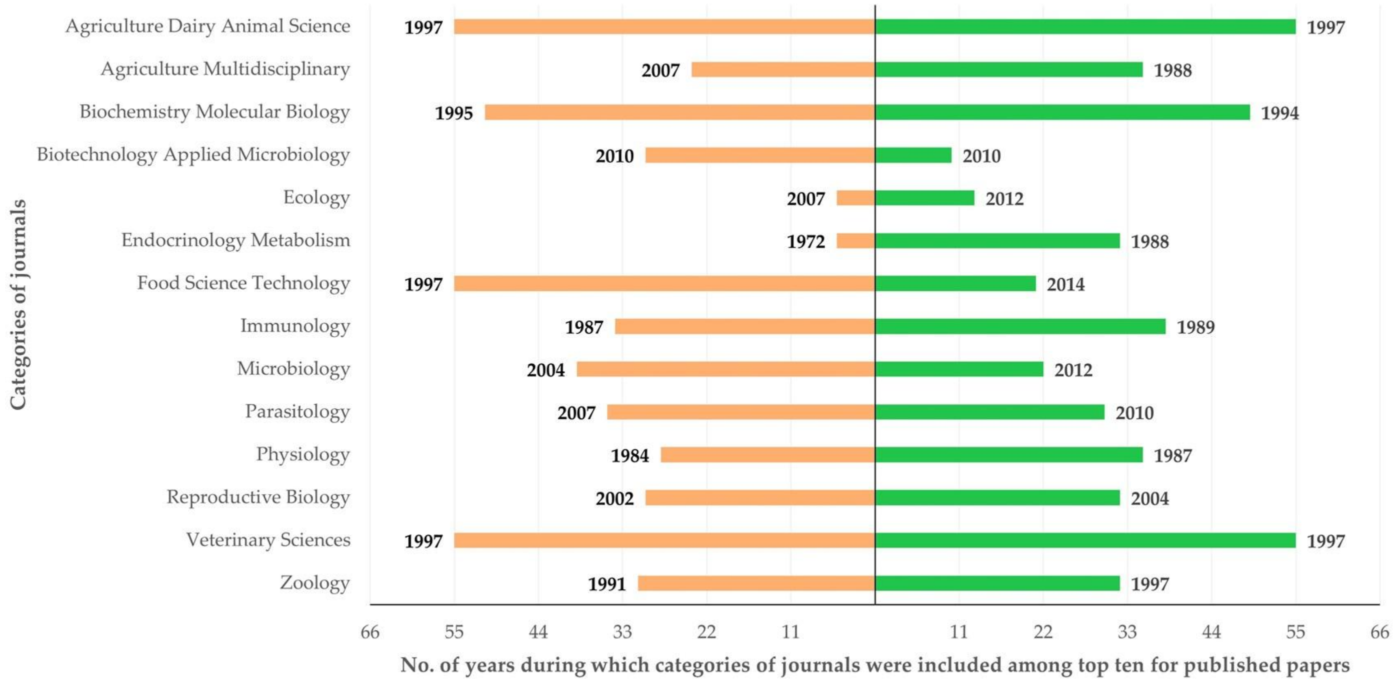

[1] Length of bars indicates the number of years during which categories of journals were included among the top ten categories for published papers.

[2] Number at edge of bars indicates the median year of publication of respective published papers during these years.

Table S9. Number of years (n) for top and second top Web of Science categories of journals on annual basis for papers related to sheep or goats published from 1970 to 2024.

Categories of Journals	Papers Related to Sheep		Papers Related to Goats	
	Years as top journal (n)	Years as second top journal (n)	Years as top journal (n)	Years as second top journal (n)
Agriculture Dairy Animal Science	0	36	4	40
Agriculture Multidisciplinary	0	5	0	0
Biochemistry Molecular Biology	0	0	0	7
Immunology	7	7	1	4
Physiology	0	0	0	1
Veterinary Sciences	48	7	50	4

Table S10. No. of years (*n*) for Web of Science categories of journals (in alphabetical order), in which most papers related to sheep or goats were published annually during the years 1970 to 1980 and 2014 to 2024.

Papers Related to Sheep			
**Period 1970-1980**		**Period 2014-2024**	
Journals	no. of years	Journals	no. of years
Agriculture Dairy Animal Science	11	Agriculture Dairy Animal Science	11
Agriculture Multidisciplinary	11	Agriculture Multidisciplinary	8
Agronomy	2	Biochemistry Molecular Biology	6
Biochemistry Molecular Biology	11	Biotechnology Applied Microbiology	2
Biology	4	Ecology	6
Biophysics	5	Environmental Sciences	9
Cell Biology	6	Food Science Technology	11
Endocrinology Metabolism	9	Genetics Heredity	1
Immunology	11	Microbiology	9
Medicine Research Experimental	9	Multidisciplinary Sciences	11
Multidisciplinary Sciences	4	Parasitology	11
Nutrition Dietetics	3	Reproductive Biology	6
Pathology	1	Veterinary Sciences	11
Physiology	10	Zoology	8
Veterinary Sciences	11		
Zoology	3		
**Papers Related to Goats**			
**Period 1970-1980**		**Period 2014-2024**	
Journals	no. of years	Journals	no. of years
Agriculture Dairy Animal Science	11	Agriculture Dairy Animal Science	11
Agriculture Multidisciplinary	4	Agriculture Multidisciplinary	5
Agronomy	1	Biochemistry Molecular Biology	7
Biochemical Research Methods	1	Biology	1
Biochemistry Molecular Biology	11	Biotechnology Applied Microbiology	11
Biology	3	Chemistry Applied	2
Biophysics	8	Ecology	1
Cell Biology	5	Environmental Sciences	4
Endocrinology Metabolism	4	Food Science Technology	11
Food Science Technology	11	Genetics Heredity	1
Immunology	11	Infectious Diseases	5
Infectious Diseases	1	Microbiology	11
Medicine General Internal	2	Multidisciplinary Sciences	10
Medicine Research Experimental	6	Parasitology	11
Multidisciplinary Sciences	6	Reproductive Biology	4
Neurosciences	2	Veterinary Sciences	11
Parasitology	1	Zoology	6
Pathology	4		
Pharmacology Pharmacy	2		
Physiology	11		
Veterinary Sciences	11		
Zoology	7		

Table S11. The languages (in order of results count), in which papers related to sheep or goats published from 1970 to 2024, were written, with respective numbers (*n*) of published papers.

Papers Related to Sheep		Papers Related to Goats	
**Language**	***n***	**Language**	***n***
English	156,995	English	64,156
German	2160	Portuguese	864
French	1609	French	763
Portuguese	1513	German	732
Spanish	699	Spanish	383
Russian	509	Polish	107
Czech	316	Chinese	106
Polish	249	Turkish	106
Turkish	238	Russian	77
Hungarian	215	Italian	75
Italian	132	Czech	59
Chinese	98	Hungarian	55
Dutch	91	Dutch	43
Japanese	64	Japanese	29
Croatian	39	Croatian	17
Ukrainian	22	Korean	14
Afrikaans	21	Norwegian	7
Romanian	14	Ukrainian	7
Slovak	10	Indonesian	6
Swedish	8	Lithuanian	5
Korean	7	Slovak	5
Norwegian	7	Afrikaans	4
Slovenian	7	Catalan	3
Lithuanian	5	Greek	3
Indonesian	4	Slovenian	2
Arabic	3	Arabic	1
Icelandic	3	Bulgarian	1
Serbo Croatian	3	Danish	1
Estonian	2	Estonian	1
Greek	2	Icelandic	1
Malay	2	Malay	1
Catalan	1	Persian	1
Danish	1	Romanian	1
Persian	1		

Table S12. The 100 countries (in order of results count), from which originated most papers related to sheep or goats published from 1970 to 2024, with respective numbers (*n*) of published papers.

Papers Related to Sheep		Papers Related to Goats	
**Country of origin**	***n***	**Country of origin**	***n***
United States of America	39,526	United States of America	12,135
United Kingdom	21304	China	7280
Australia	15,606	India	6944
China	9271	Spain	4085
France	9162	United Kingdom	4026
Germany	8952	Brazil	3892
Brazil	6718	France	3671
Canada	6701	Italy	3106
Spain	6641	Germany	2888
Italy	6135	Japan	2225
New Zealand	5921	Australia	2057
India	5917	Turkiye	2051
Japan	4985	The Netherlands	1681
Turkiye	3938	Canada	1579
Iran	3247	Iran	1418
The Netherlands	3104	South Africa	1332
Switzerland	2898	Mexico	1302
Poland	2590	Egypt	1204
South Africa	2472	Pakistan	1179
Sweden	2237	Switzerland	1122
Belgium	2211	Nigeria	997
Mexico	2139	Greece	949
Norway	1868	Poland	906
Greece	1741	Belgium	805
Egypt	1632	Kenya	804
Argentina	1601	Sweden	783
Pakistan	1367	New Zealand	778
Denmark	1355	Saudi Arabia	742
Czechia	1353	Ethiopia	717
Austria	1343	Norway	705
Israel	1321	Israel	620
Ireland	1254	Argentina	608
Russian Federation	1141	Portugal	590
Ethiopia	1112	Republic of Korea	585
Saudi Arabia	999	Malaysia	575
Portugal	979	Denmark	565
Hungary	905	Austria	499
Nigeria	826	Thailand	449
Republic of Korea	819	Czechia	420
Finland	789	Russian Federation	339
Uruguay	772	Indonesia	334
Kenya	760	Sudan	304
Slovakia	733	Bangladesh	298
Chile	676	Tunisia	291
Tunisia	568	Tanzania	287
Romania	536	Hungary	274
Malaysia	511	Taiwan	271
Jordan	449	Ireland	266
Morocco	434	Cote d' Ivoire	255
Iraq	421	Jordan	249
Croatia	404	Slovakia	246
Thailand	358	Romania	239
Algeria	348	Finland	237
Bulgaria	343	Algeria	236

Sudan	325	Morocco	230
Taiwan	320	Chile	228
Serbia	319	Iraq	215
Indonesia	299	Uruguay	196
Colombia	276	Uganda	169
Slovenia	234	Serbia	160
Tanzania	222	United Arab Emirates	154
Bangladesh	218	Zimbabwe	142
Iceland	211	Cyprus	140
United Arab Emirates	210	Ghana	138
Venezuela	189	Bulgaria	134
Peru	182	Senegal	131
Senegal	174	Oman	125
Singapore	173	Venezuela	120
Kazakhstan	170	Colombia	119
Cyprus	167	Vietnam	117
Zimbabwe	166	Mongolia	102
Ghana	165	Slovenia	102
Syria	159	Nepal	98
Ukraine	143	Cameroon	89
Kuwait	130	Philippines	88
Vietnam	127	Singapore	84
Mongolia	122	Ecuador	82
Oman	120	Zambia	78
Uganda	119	Sri Lanka	77
Cuba	116	Peru	72
Burkina faso	115	Botswana	71
Lebanon	100	Burkina Faso	71
Estonia	98	Lebanon	71
Lithuania	98	Syria	71
Cameroon	92	Kazakhstan	66
Nepal	92	Mozambique	63
Ecuador	79	Croatia	62
Bosnia and Herzegovina	72	Lithuania	60
Libya	69	Ukraine	54
Costa Rica	66	Costa Rica	49
Sri Lanka	64	Namibia	48
Saint Kitts and Nevis	63	Saint Kitts and Nevis	47
Cote d' Ivoire	62	Benin	45
Philippines	60	Bosnia and Herzegovina	45
Zambia	60	Libya	43
Benin	58	Malawi	43
Kyrgyzstan	58	Gambia	41
Namibia	57	Trinidad and Tobago	36
Trinidad and Tobago	57	Lao People's Democratic Republic	33
Botswana	56	Yemen	33
Niger	56		

Figure S6. Filled maps of countries, from which originated at least 1% of papers related to sheep (a) or to goats (b) published from 1970 to 2024, in accordance with respective number of papers.

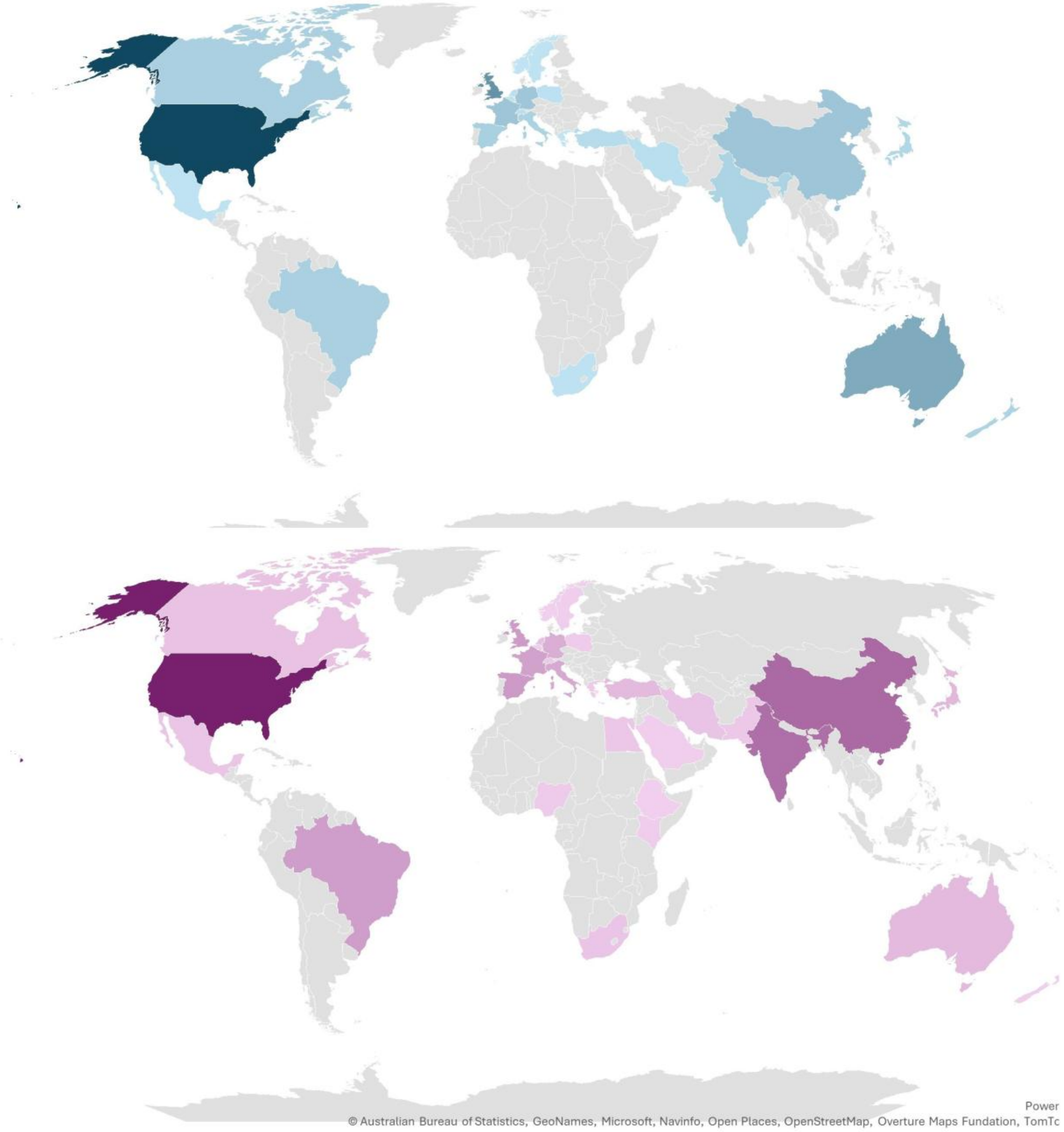

(a)

(b)

Table S13. Numbers of papers (*n*) related to sheep or to goats published from 1970 to 2024, in accordance with the geographical subregions of the countries of origin and respective proportions (%) among all relevant papers.

Geographical Subregions	Published Papers			
	Related to Sheep		Related to Goats	
	n	%	*n*	%
Australia and New Zealand	21,527	13.0	2835	4.2
Caribbean	277	0.2	117	0.2
Central America	2244	1.4	1376	2.0
Central Asia	283	0.2	127	0.2
Eastern Africa	2601	1.6	2430	3.6
Eastern Asia	15,521	9.4	10,463	15.5
Eastern Europe	7786	4.7	2629	3.9
Melanesia	20	0.0	18	0.0
Micronesia	1	0.0	1	0.0
Middle Africa	168	0.1	185	0.3
Northern Africa	3376	2.0	2308	3.4
Northern America	46,227	28.0	13,714	20.3
Northern Europe	29,253	17.7	6717	9.9
Polynesia	6	0.0	18	0.0
South America	10,550	6.4	5333	7.9
South-eastern Asia	1571	1.0	1711	2.5
Southern Africa	2613	1.6	1466	2.2
Southern Asia	10,957	6.6	10,049	14.9
Southern Europe	16,667	10.1	9179	13.6
Western Africa	1603	1.0	1790	2.6
Western Asia	8186	5.0	4586	6.8
Western Europe	27,700	16.8	10,676	15.8

Figure S7. 'Butterfly'-type plot of years [1] (n) of inclusion of countries of origin among the top ten for papers related to sheep (green) or to goats (coral) published from 1970 to 2024 (year at edge of bar indicates median year of publication of respective published papers [2]).

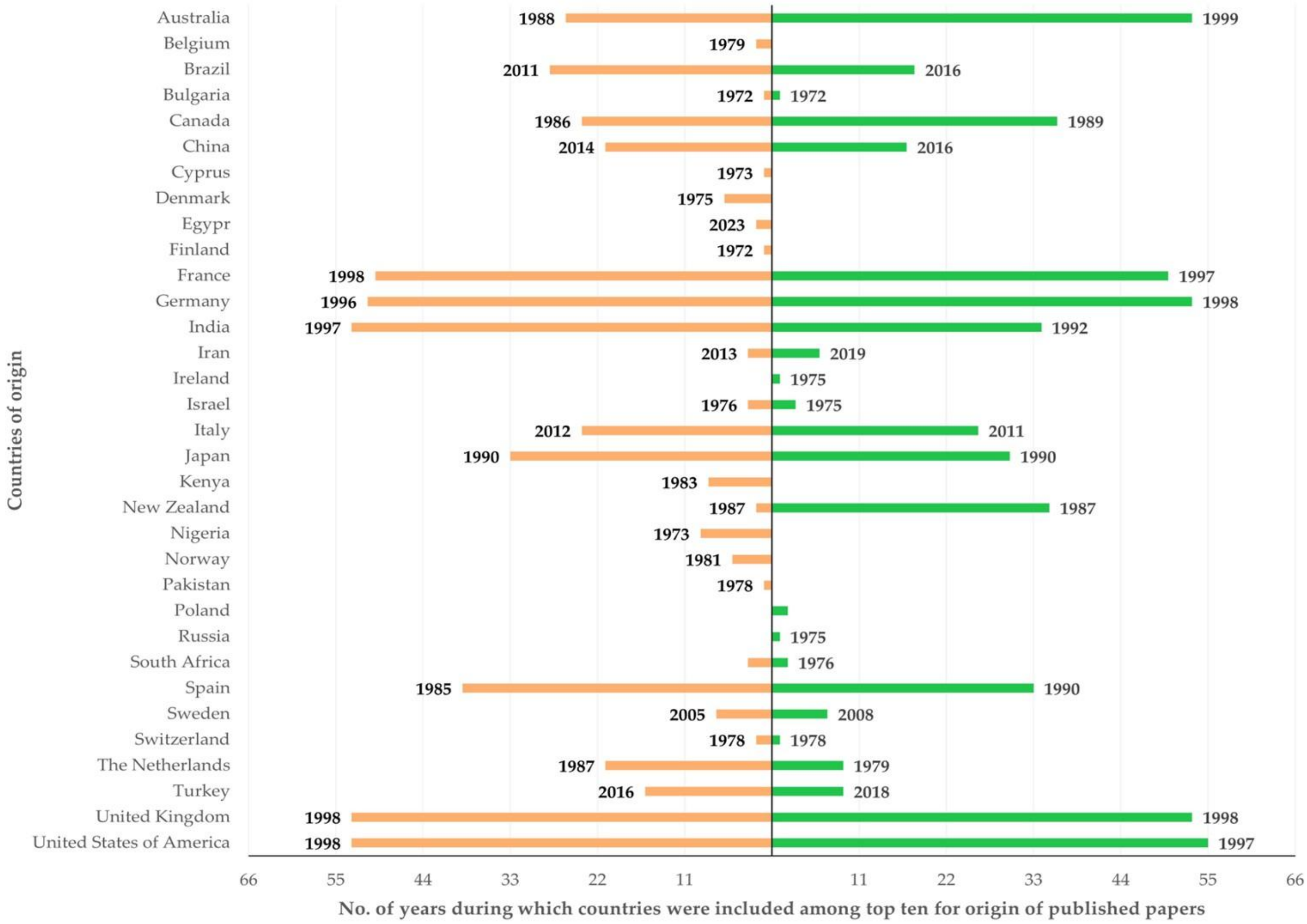

[1] Length of bars indicates the number of years during which countries of journals were included among the top ten countries for origin of published papers.

[2] Number at edge of bars indicates the median year of publication of respective published papers during these years.

Table S14. Number of years (*n*) for top and second top countries of origin on annual basis for papers related to sheep or goats published from 1970 to 2024.

Countries	Papers related to sheep		Papers related to goats	
	Years as top journal (*n*)	Years as second top journal (*n*)	Years as top journal (*n*)	Years as second top journal (*n*)
Australia	0	2	0	0
Canada	0	1	1	0
China	3	4	11	3
India	0	0	1	34
United Kingdom	0	44	0	4
United States of America	52	3	41	12

Table S15. Number of years (*n*) for countries of origin (in alphabetical order), for most papers related to sheep or goats published annually during the years 1970 to 1980 and 2014 to 2024.

**Papers Related to Sheep**			
**Period 1970-1980**		**Period 2014-2024**	
Countries	no. of years	Countries	no. of years
Australia	9	Australia	11
Bulgaria	1	Brazil	11
Canada	10	China	11
France	9	France	8
Germany	9	Germany	11
India	6	India	3
Ireland	1	Iran	5
Israel	3	Italy	11
Italy	2	New Zealand	1
Japan	6	Spain	11
New Zealand	9	Turkey	5
Poland	2	United Kingdom	11
Russia	1	United States of America	11
Sweden	5		
Switzerland	1		
United Kingdom	9		
United States of America	11		
**Papers Related to Goats**			
**Period 1970-1980**		**Period 2014-2024**	
Countries	no. of years	Countries	no. of years
Australia	6	Australia	1
Belgium	1	Brazil	11
Bulgaria	1	China	11
Canada	8	Egypt	2
Cyprus	1	France	8
Denmark	5	Germany	11
Finland	1	India	11
France	8	Iran	1
Germany	9	Italy	10
India	9	Pakistan	1
Israel	2	Spain	11
Japan	7	Turkiye	10
Kenya	4	United Kingdom	11
New Zealand	2	United States of America	11
Nigeria	4		
Norway	4		
South Africa	1		
Sweden	4		
The Netherlands	5		
United Kingdom	9		
United States of America	6		

Table S16. The 100 scientific organisations (in order of results count), with which most papers related to sheep or goats published from 1970 to 2024 were affiliated, with respective numbers (*n*) of published papers.

Papers Related to Sheep		Papers Related to Goats	
**Scientific organisations**	***n***	**Scientific organisations**	***n***
Institut National de Recherche pour l' Agriculture, l' Alimentation et l' Environnement	4600	Indian Council of Agricultural Research	2538
Commonwealth Scientific Industrial Research Organisation	3108	Institut National de Recherche pour l' Agriculture, l' Alimentation et l' Environnement	2040
United States Department of Agriculture	3070	United States Department of Agriculture	1183
Indian Council of Agricultural Research	2286	Northwest A F University	1123
University of Edinburgh	2275	Indian Veterinary Research Institute	1082
University of Melbourne	2255	Consejo Superior de Investigaciones Cientificas	794
Centre National de la Recherche Scientifique	2101	Centre National de la Recherche Scientifique	645
AgResearch New Zealand	2069	University of California Davis	627
Monash University	1474	Universite Paris Saclay	538
University of Sydney	1424	Utrecht University	526
Harvard University	1404	Universidade Estadual Paulista	474
Massey University	1390	Autonomous University of Barcelona	456
University of California Davis	1382	University of Pretoria	456
Adelaide University	1338	Texas A M University	453
Moredun Research Institute	1298	Universidad de Cordoba	435
Department of Primary Industries Regional Development New South Wales	1294	Universidade de Sao Paulo	417
Consejo Superior de Investigaciones Cientificas	1232	Norwegian University of Life Sciences	404
Institut National de la Sante et de la Recherche Medicale	1226	Nanjing Agricultural University	398
Texas A M University	1222	Swedish University of Agricultural Sciences	392
University of Adelaide	1222	Universidade Federal Rural de Pernambuco	387
Universite Paris Saclay	1124	Aristotle University of Thessaloniki	384
National Institutes of Health United States of America	1116	Universidade Estadual do Ceara	384
University of Cambridge	1066	International Livestock Research Institute	377
University of Aberdeen	1060	Washington State University	374
University of Western Australia	1058	University of Milan	372
University of Queensland	1053	Universidade Federal de Campina Grande	368
University of New England	1034	University of Bern	368
Colorado State University Fort Collins	1008	Universidad Nacional Autonoma de Mexico	355
University of California San Francisco	981	Complutense University of Madrid	337
Universidade Estadual Paulista	970	University of Zurich	337
Universidade de Sao Paulo	965	National Institutes of Health United States of America	335
University of Florida	948	Wageningen University Research	334
Cornell University	940	Universidade Federal da Paraiba	332
Indian Veterinary Research Institute	857	King Saud University	331
University of Zurich	852	University of Edinburgh	330
Universite Paris Cite	847	Universiti Putra Malaysia	329
University of Nottingham	840	University of Copenhagen	324
University of Glasgow	839	Cornell University	323
University of California Los Angeles	836	Commonwealth Scientific Industrial Research Organisation	310
University of Auckland	828	Universite de Toulouse	308
Norwegian University of Life Sciences	826	Harvard University	305
University of Wisconsin Madison	826	Institut National de la Sante et de la Recherche Medicale	303
Universite de Toulouse	815	University of Sassari	302
University of Sassari	789	University of Agriculture Faisalabad	301
Roslin Institute	776	University of Murcia	298
University of Bristol	772	University of Florida	293
University of Otago	763	University of Veterinary Animal Science Pakistan	293
United States Department of Veterans Affairs	758	University of Queensland	291

Washington State University	758	Universidad de las Palmas de Gran Canaria	287
University of Guelph	754	University of Tokyo	284
Wageningen University Research	754	University of Wisconsin Madison	278
University of Michigan	747	Centre International de Recherche Agronomique pour la Development	273
University of Liverpool	745	China Agricultural University	270
University of Zaragoza	744	University of Veterinary Medicine Hannover	267
University of Pennsylvania	739	Cairo University	263
University of Bern	725	Justus Liebig University Giessen	262
University of Pretoria	724	Islamic Azad University	259
Universidad de Leon	716	Universidade Federal de Minas Gerais	258
Aristotle University of Thessaloniki	705	Yangzhou University	257
Utrecht University	705	Vetagro SUP	255
Swedish University of Agricultural Sciences	704	Friedrich Loeffler Institute	254
Babraham Institute	697	Universita degli Studi di Bari Aldo Moro	253
Agriculture Agri Food Canada	684	University of Zaragoza	253
China Agricultural University	684	University of Illinois Urbana Champaign	252
Free University of Berlin	682	Universidade Federal de Vicosa	251
University of Toronto	682	Ankara University	249
University College London	668	Ecole Nationale Veterinaire de Toulouse	249
University of Copenhagen	666	Agence Nationale de Securite Sanitaire de l′ Alimentation de l′ Environnement du Travail	248
Imperial College London	655	AgroParisTech	247
Consejo Nacional de Investigaciones Cientificas y Tecnicas	652	Universidade Federal da Bahia	243
James Hutton Institute	648	University of Guelph	243
University of Veterinary Medicine Hannover	641	AgResearch New Zealand	242
Ecole Nationale Veterinaire de Toulouse	635	Langston University	242
Justus Liebig University Giessen	626	Pirbright Institute	235
Complutense University of Madrid	625	Massey University	232
Pirbright Institute	624	University of Naples Federico ii	228
University of Minnesota Twin Cities	614	Lanzhou Veterinary Research Institute	227
University College Dublin	603	University of Turin	224
Murdoch University	598	Tamil Nadu Veterinary Animal Sciences University	223
Virginia Polytechnic Institute State University	597	Consejo Nacional de Investigaciones Cientificas y Tecnicas	220
International Livestock Research Institute	591	Botswana Ministry of Agriculture	219
University of New South Wales Sydney	589	Huazhong Agricultural University	219
Lincoln University New Zealand	586	University of Sydney	219
University of Missouri	586	University of Nairobi	217
University of Alberta	580	Sichuan Agricultural University	216
University of Missouri Columbia	567	Universidade Federal do Ceara	213
Ohio State University	562	University of Tehran	212
Universidad Nacional Autonoma de Mexico	560	Universidad de Leon	210
Islamic Azad University	555	Radboud University Nijmegen	209
University of Wyoming	550	Ghent University	208
Utah State University	543	University of Liege	207
Central Sheep and Wool Research Institute	541	University of Minnesota Twin Cities	207
The Royal Veterinary College London	539	Institute of Animal Science China	202
Hudson Institute of Medical Research	519	Colorado State University Fort Collins	201
McGill University	513	United States Department of Veterans Affairs	201
Autonomous University of Barcelona	511	University of Veterinary Medicine Vienna	200
Gansu Agricultural University	507	Assam Agricultural University	197
Scottish Agricultural College	506	Addis Ababa University	195
University of Munich	503	Free University of Berlin	194
University of Liege	501	Food and Agriculture Organization of the United Nations	191

Table S17. List of countries (in order of results count) with the 100 scientific organisations with most papers related to sheep or to goats published from 1970 to 2024, with respective number (*n*) of scientific organisations.

Papers Related to Sheep		Papers Related to Goats	
**Countries of location of scientific organisations**	***n***	**Countries of location of scientific organisations**	***n***
United States	22	United States	14
United Kingdom	15	Brazil	10
Australia	13	France	10
France	7	Spain	9
Canada	6	China	8
Spain	6	Italy	6
New Zealand	5	India	4
Germany	4	Australia	3
India	3	Germany	3
Brazil	2	The Netherlands	3
China	2	Belgium	2
Switzerland	2	Greece	2
The Netherlands	2	Iran	2
Belgium	1	Kenya	2
Denmark	1	New Zealand	2
Greece	1	Pakistan	2
Iran	1	Switzerland	2
Ireland	1	United Kingdom	2
Italy	1	Austria	1
Kenya	1	Botswana	1
Mexico	1	Canada	1
Norway	1	Denmark	1
South Africa	1	Egypt	1
Sweden	1	Ethiopia	1
		Japan	1
		Malaysia	1
		Mexico	1
		Norway	1
		Saudi Arabia	1
		South Africa	1
		Sweden	1
		Turkiye	1

Table S18. The 100 topics-meso (in order of results count) of most papers related to sheep or goats published from 1970 to 2024, with respective numbers (*n*) of published papers.

Papers Related to Sheep		Papers Related to Goats	
**Topics-meso**	***n***	**Topics-meso**	***n***
Dairy & Animal Sciences	33,137	Dairy & Animal Sciences	13,436
Parasitology - General	10,635	Food Science & Technology	3385
Immunology	5701	Parasitology - General	3284
Reproductive Biology	5526	Reproductive Biology	2864
Obstetrics & Gynecology	4726	Zoonotic Diseases	2754
Zoonotic Diseases	4126	Virology - General	1457
Zoology & Animal Ecology	3870	Inflammatory Bowel Diseases & Infections	1388
Endocrinology & Metabolism	3502	Zoology & Animal Ecology	1214
Neurodegenerative Diseases	2767	Sexually Transmitted Infections	1187
Bacteriology	2432	Parasitology - Malaria, Toxoplasmosis & Coccidiosis	1127
Virology - General	2229	Immunology	1085
Cardiology - General	2209	*Human Immunodeficiency Virus*	984
Neuroscience	2208	Virology - Identification & Sequencing	976
Virology - Tropical Diseases	2207	Antibiotics & Antimicrobials	953
Forestry	2107	Virology - Tropical Diseases	947
Food Science & Technology	2100	Archaeology	870
Sexually Transmitted Infections	2087	Bacteriology	834
Parasitology - Malaria, Toxoplasmosis & Coccidiosis	1835	Forestry	799
Virology - Identification & Sequencing	1803	Phylogenetics & Genomics	716
Orthopedics	1791	Tuberculosis & Leprosy	663
Soil Science	1702	Endocrinology & Metabolism	591
Antibiotics & Antimicrobials	1683	Neurodegenerative Diseases	589
Assisted Ventilation	1661	Veterinary Sciences	573
Entomology	1478	Diabetes	556
Stem Cell Research	1468	Parasitology - *Trypanosoma* & *Leishmania*	556
Human Immunodeficiency Virus	1458	Stem Cell Research	525
Molecular & Cell Biology - Physiology	1371	Diarrheal Diseases	511
Archaeology	1360	Herbicides, Pesticides & Ground Poisoning	474
Anesthesiology	1356	Phytochemicals	454
Physiology & Metals	1323	Contamination & Phytoremediation	448
Contamination & Phytoremediation	1281	Orthopedics	441
Phylogenetics & Genomics	1167	Lipids	439
Molecular Toxicology	1125	Anesthesiology	431
Sleep Science & Circadian Systems	1106	Agricultural Policy	408
Diabetes	1083	Physiology & Metals	402
Inflammatory Bowel Diseases & Infections	1024	Chromosome Disorders	397
Extracellular Matrix & Cell Differentiation	1008	Molecular & Cell Biology - Genetics	394
Herbicides, Pesticides & Ground Poisoning	994	Chemometrics	387
Urology & Nephrology - General	941	Molecular & Cell Biology - Immunotherapy	381
Molecular & Cell Biology - Pharmacology	935	Bacterial Toxins & Diseases	368
Phytochemicals	931	Cardiology - General	368
Tuberculosis & Leprosy	928	Genome Studies	352
Dentistry & Oral Medicine	907	Extracellular Matrix & Cell Differentiation	345
Neuroendocrine & Intestinal Disorders	874	Biosensors	344
Molecular & Cell Biology - Genetics	871	Dermatology - General	337
Veterinary Sciences	844	Entomology	334
Bone Diseases	836	Allergy	330
Agricultural Policy	786	Soil Science	327

Molecular & Cell Biology - Immunotherapy	779
Chromosome Disorders	767
Nanofibers, Scaffolds & Fabrication	755
Cardiac Arrhythmia	711
Diarrheal Diseases	705
Genome Studies	681
Immunology & Hematology	680
Complementary & Alternative Medicine	640
Back Pain	615
Lipids	594
Allergy	559
Hormone Therapy	550
Parasitology - *Trypanosoma* & *Leishmania*	542
Tissue Barriers	534
Mycotoxins	522
Blood Clotting	508
Pharmacology & Toxicology	506
Bacterial Toxins & Diseases	493
Blood Disorders	489
Chemometrics	488
Marine Biology	458
Hematologic Diseases	438
Substance Abuse	415
Microbial Biotechnology	412
Membrane Channels & Receptors	410
Thyroid Disorders	395
Dermatology - General	393
Musculoskeletal Disorders	392
Birth Defects	378
Crop Science	373
Molecular & Cell Biology - Cancer, Autophagy & Apoptosis	364
Cardiology - Circulation	359
Micro & Long Noncoding Rna	353
Ophthalmology	353
Synthesis	343
Wounds & Ulcers	342
Bioengineering	330
Drug Delivery Chemistry	328
Autonomic Regulation	320
Abdominal Surgery	306
Vitamin Metabolism	281
Lymphomas	274
Plant Pathology	272
Cell Biology	256
Gastrointestinal & Esophageal Diseases	255
Digestive System Disorders	254
Lysosomal Storage Disorders	253
Surfactants, Lipid Bilayers & Antimicrobial Peptides	250
Medical Mycology	237
Cosmetic Surgery	232
Molecular & Cell Biology - Mitochondria	219

Molecular & Cell Biology - Cancer, Autophagy & Apoptosis	313
Micro & Long Noncoding Rna	310
Marine Biology	308
Microbial Biotechnology	305
Nanofibers, Scaffolds & Fabrication	289
Neuroscience	280
Dentistry & Oral Medicine	266
Autonomic Regulation	263
Crop Science	259
Mycotoxins	258
Pharmacology & Toxicology	249
Bone Diseases	244
Drug Delivery Chemistry	242
Blood Clotting	239
Complementary & Alternative Medicine	239
Back Pain	220
Cardiac Arrhythmia	218
Tissue Barriers	215
Sleep Science & Circadian Systems	205
Hematologic Diseases	198
Crop Protection	186
Smell & Taste Science	186
Hormone Therapy	183
Obstetrics & Gynecology	183
Molecular & Cell Biology - Physiology	181
Nanoparticles	173
Synthesis	172
Thyroid Disorders	165
Molecular & Cell Biology - Pharmacology	164
Musculoskeletal Disorders	164
Molecular Toxicology	154
Urology & Nephrology - General	146
Ophthalmology	141
Bioengineering	134
Chromatography & Electrophoresis	132
Medical Mycology	131
Cell Biology	126
Polymers & Macromolecules	125
Birth Defects	124
Sports Science	123
Lysosomal Storage Disorders	122
Neuroendocrine & Intestinal Disorders	117
Protein Structure, Folding & Modelling	109
Immunology & Hematology	107
Plant Pathology	102
Ancient Religion & Literature	98
Assisted Ventilation	95
Blood Disorders	91
Paper & Wood Materials Science	89
Trauma & Emergency Surgery	89
Molecular & Cell Biology - Mitochondria	87

Smell & Taste Science	204	Membrane Channels & Receptors	86

Table S19. The 100 topics-micro (in order of results count) of most papers related to sheep or goats published from 1970 to 2024, with respective numbers (*n*) of published papers.

Papers Related to Sheep		Papers Related to Goats	
**Topics-micro**	***n***	**Topics-micro**	***n***
Ruminant nutrition	15,323	Ruminant nutrition	5668
Livestock reproduction	8656	Livestock reproduction	3357
Anthelmintic resistance	7130	Dairy product chemistry	2915
Farm animal welfare	3691	Anthelmintic resistance	2278
Wildlife ecology	2822	Farm animal welfare	1233
Prion pathogenesis	2658	Mastitis	1195
Meat quality	2539	Meat quality	1115
Embryo development	2102	Tick-borne pathogens	1082
Tick-borne pathogens	1998	Male fertility	1047
Dairy product chemistry	1673	Wildlife ecology	959
Echinococcosis	1663	Lactic acid bacteria	955
Neonatal hypoxia effects	1659	Embryo development	939
Somatic hypermutation	1584	*Human immunodeficiency virus* pathogenesis	929
Maternal-fetal health	1580	Brucellosis	875
Gonadotropin-releasing hormone (GnRH)	1576	*Toxoplasma gondii*	822
Schistosomiasis	1551	*Mycoplasma* infections	740
Toxoplasma gondii	1428	Viral disease dynamics	717
Arbovirus dynamics	1351	Tuberculosis diagnostics and treatment	660
Growth hormone / Insulin Growth Factor axis	1275	*Coxiella burnetii*	644
Stress and cortisol	1206	*Poxvirus* immunology	616
Brucellosis	1174	Genetic diversity	611
T cell regulation	1057	Prion pathogenesis	535
Mastitis	1053	Livestock fiber traits	518
Interferons	1033	*Trypanosoma* biology	470
Circadian-melatonin	1024	Fertility preservation	451
Genetic diversity	1003	Echinococcosis	450
Human immunodeficiency virus pathogenesis	997	*Arbovirus* dynamics	424
Fertility preservation	988	Holocene	419
Male fertility	975	Methicillin-resistant *Staphylococcus aureus* and Vancomycin-resistant *Enterococcus*	382
Plant communities	959	Schistosomiasis	381
Zinc nutrition	939	Somatic hypermutation	361
Mechanical ventilation	928	*Corynebacterium* infections	357
Tuberculosis diagnostics and treatment	902	*Cryptosporidium*	348
Poxvirus immunology	874	Appetite hormones	334
Livestock fiber traits	839	Spermatogenesis	320
Escherichia coli pathogenesis	833	Viral hemorrhagic fevers	314
Forensic entomology	828	Antibiotic pharmacokinetics	313
Pasteurella multocida	819	Rangeland dynamics	310
Rangeland dynamics	736	Mesenchymal stem cells	308
Coxiella burnetii	700	Population genetics	304
Poultry nutrition	697	Plant communities	280
Mesenchymal stem cells	691	Veterinary anesthesia	274
Valve interventions	681	Nanobiosensors	272
Complement system	678	Sustainable agriculture	268
Preterm birth causes	671	Colostrum	265
Selenium	660	Molecular authentication	260
Mycoplasma infections	642	Herpesvirus dynamics	259
Spermatogenesis	642	*Pasteurella multocida*	259
Livestock viral threats	638	Respiratory control	254
Holocene	628	*Enterovirus* research	251
Preeclampsia factors	627	Growth hormone / Insulin Growth Factor axis	249
Salmonella and *Campylobacter*	614	Hydroxyapatite composites	249
Hydroxyapatite composites	609	Hair disorders	247

Viral disease dynamics	604	Functional bioactive peptides	247
Pyrrolizidine alkaloids	596	Gonadotropin-releasing hormone (GnRH)	243
Fracture management	586	*Escherichia coli* pathogenesis	242
Population genetics	569	Livestock viral threats	229
Chlamydia infections	560	Zinc nutrition	225
Prolactin	558	Collagen disorders	224
Tissue engineering	549	Coccidiosis	224
Methicillin-resistant *Staphylococcus aureus* and Vanco-mycin-resistant *Enterococcus*	542	Equine and bovine gastrointestinal disorders	223
Glycosylation roles	540	Pyrrolizidine alkaloids	214
Appetite hormones	535	Mycotoxin control	211
Viral hemorrhagic fevers	533	*Clostridium* infections	210
Renin-angiotensin System	524	Oral delivery	206
Soil carbon dynamics	522	Gut microbiota	205
Sustainable agriculture	519	Chromosomal evolution	201
Epithelial ion transport	494	Mosquito-borne viruses	199
Corynebacterium infections	481	Stable isotopes	197
Spinal disorders	472	Circadian-melatonin	191
Enterovirus research	470	*Listeria monocytogenes*	191
Chromosomal evolution	464	Allergy mechanisms	185
Herpesvirus dynamics	456	Solid-phase microextraction	184
Transposable elements	447	Mechanical circulatory support	182
Oxytocin and vasopressin	444	Selenium	182
Trypanosoma biology	439	Anterior cruciate ligament	179
Pulmonary surfactant	434	*Salmonella* and *Campylobacter*	178
Lipoxygenase pathways	428	Veterinary oncology	177
Veterinary anesthesia	428	Atrial fibrillation management	173
Anterior cruciate ligament	420	Glycosylation roles	171
Cryptosporidium	416	Fatty acids	167
Sepsis immunology	409	Herbicide resistance	166
Osteoporosis	399	T cell regulation	165
Clostridium infections	395	Forensic entomology	164
Atrial fibrillation management	386	Lead and cadmium toxicity	164
Thyroid disorders	385	Sex chromosome variations	163
Lactic acid bacteria	384	Microrna in cancer	163
Listeria monocytogenes	382	Spinal disorders	161
Stable isotopes	380	Cytochrome P450	160
Neuropeptide roles	378	*Chlamydia* infections	160
Nitrogen management	377	Thyroid disorders	155
Gestational trophoblastic disease	362	Leptospirosis epidemiology	153
Human T-lymphotrophic virus type 1 / Bovine leucaemia virus pathogenesis	357	Transposable elements	131
Mechanical circulatory support	340	Enteric viruses	121
Antibiotic pharmacokinetics	337	Cultural archaeology	121
Molecular authentication	337	Enzyme replacement therapy	118
Nitric oxide roles	332	Mammalia	117
Lead and cadmium toxicity	332	Protein-stabilized Emulsions	116
NaDPH Oxidase	331	Quantitative trait locus	115
Opioid receptors	329	Iminosugar synthesis	113

Figure S8. 'Butterfly'-type plot of years [1] (*n*) of inclusion of topics-micro among the top ten for papers related to sheep (green) or to goats (coral) published from 1970 to 2024 (year at edge of bar indicates median year of publication of respective papers [2]).

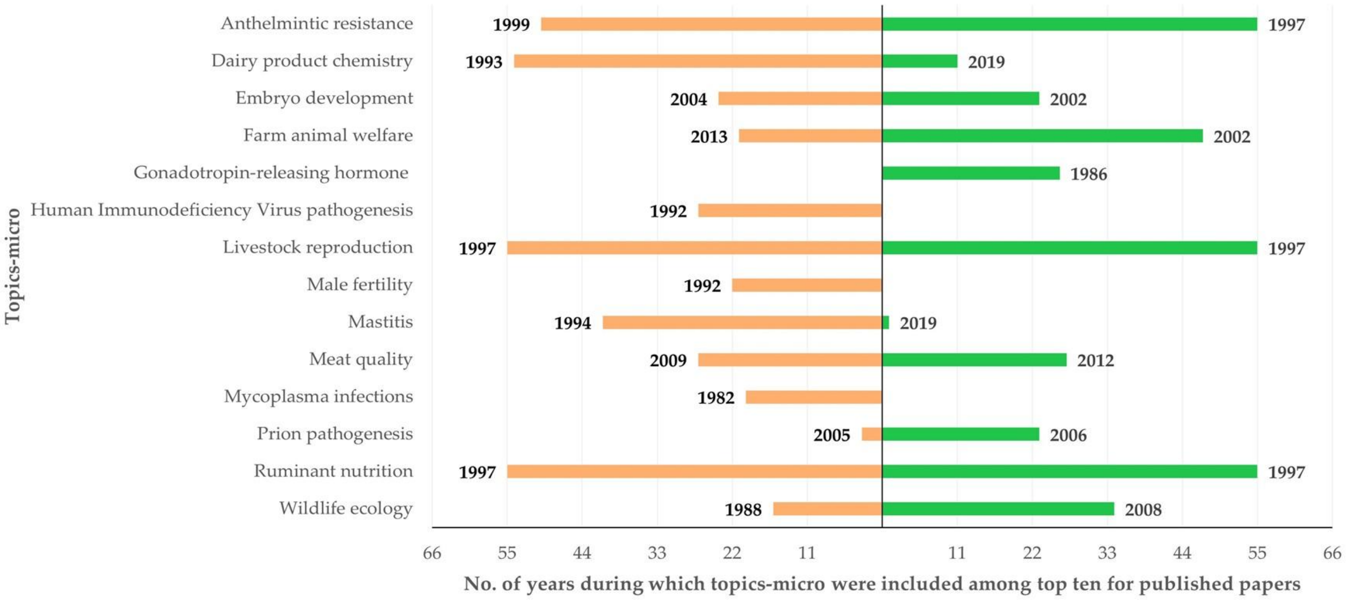

[1] Length of bars indicates the number of years during which topics-micro were included among the top ten topics-micro for published papers.

[2] Number at edge of bars indicates the median year of publication of respective published papers during these years.

Table S20. Number of years (*n*) for topics-micro (in alphabetical order), in which most papers related to sheep or goats referred to annually during the years 1970 to 1980 and 2014 to 2024.

Papers Related to Sheep			
**Period 1970-1980**		**Period 2014-2024**	
Topics-micro	no. of years	Topics-micro	no. of years
Anthelmintic resistance	11	Anthelmintic resistance	11
Cell regulation	6	*Arbovirus* dynamics	3
Chromosomal evolution	1	Brucellosis	1
Complement system	4	Dairy product chemistry	7
Embryo development	1	Echinococcosis	9
Epithelial ion transport	1	Embryo development	2
Farm animal welfare	8	Farm animal welfare	11
Gestational trophoblastic disease	1	Genetic diversity	1
Glycosylation roles	1	Livestock reproduction	11
Gonadotropin-releasing hormone (GnRH)	9	Mastitis	1
Interferons	9	Maternal-fetal health	1
Livestock fiber traits	7	Meat quality	11
Livestock reproduction	11	Population genetics	2
Meat quality	3	Prion pathogenesis	4
Neonatal hypoxia effects	2	Ruminant nutrition	11
Preeclampsia factors	1	Tick-borne pathogens	11
Prolactin	1	*Toxoplasma gondii*	5
Renin-angiotensin System	1	Wildlife ecology	11
Ruminant nutrition	11		
Schistosomiasis	3		
Somatic hypermutation	9		
Spermatogenesis	1		
Thyroid disorders	1		
Toxoplasma gondii	1		
Zinc nutrition	9		

Papers Related to Goats			
**Period 1970-1980**		**Period 2014-2024**	
Topics-micro	no. of years	Topics-micro	no. of years
Anthelmintic resistance	6	Anthelmintic resistance	11
Antibiotic pharmacokinetics	2	Brucellosis	4
Antigen retrieval	1	Coxiella *burnetii*	3
Brucellosis	3	Dairy product chemistry	11
Chromosomal evolution	2	Embryo development	2
C-myc	2	Farm animal welfare	10
Complement system	1	Genetic diversity	1
Dairy product chemistry	10	Lactic acid bacteria	11
Echinococcosis	1	Livestock reproduction	11
Farm animal welfare	3	Male fertility	3
Forensic entomology	1	Mastitis	6
Growth hormone / Insulin Growth Factor axis	2	Meat quality	9
Glycosylation roles	1	Population genetics	1
Hemoglobin function	1	Poxvirus immunology	2
Herpesvirus dynamics	1	Ruminant nutrition	11
Human immunodeficiency virus pathogenesis	3	Tick-borne Pathogens	8
Htlv-1/blv Pathogenesis	1	*Toxoplasma gondii*	1
Iminosugar synthesis	1	Viral disease dynamics	4
Interferons	1	Wildlife ecology	3
Lipoprotein metabolism	1		
Livestock fiber traits	1		
Livestock reproduction	11		
Male fertility	2		
Mastitis	8		
Maternal-fetal health	1		

Meat quality	2
Mycoplasma infections	8
Oxytocin and vasopressin	8
Peroxisomes	1
Poxvirus immunology	1
Prolactin	1
Protein folding	1
Pulmonary hypertension	1
Radiation genotoxicity	1
Radioactive contamination	1
Radioimmunotherapy	1
Renin-angiotensin system	1
Respiratory control	1
Ruminant nutrition	11
Schistosomiasis	2
Sepsis immunology	1
Sexual dysfunction	1
Sickle cell disease	5
Snake venom biochemistry	1
Somatic hypermutation	8
T cell regulation	1
Tuberculosis diagnostics & treatment	1
Thyroid disorders	1
Tick-borne pathogens	2
Toxic alcohols	1
Toxoplasma gondii	1
Trypanosoma biology	5
Veterinary anesthesia	2
Viral disease dynamics	1
Wildlife ecology	5

Figure S9. Yearly proportion (%) of papers related to sheep (green) or goats (coral) published from 1970 to 2004 under open-access mode.

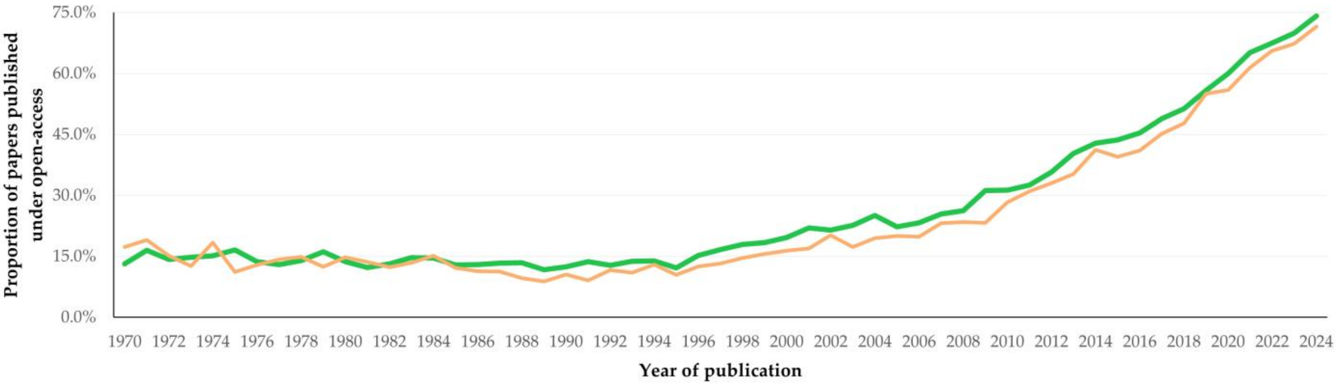

Table S21. Sustainable development goals (in order of results count) served in papers related to sheep or goats published from 1970 to 2024, with respective number (*n*) of published papers.

**Papers Related to Sheep**		**Papers Related to Goats**	
**Sustainable development goals**	***n***	**Sustainable development goals**	***n***
03.Good health and well being	116,004	03.Good health and well being	45,394
13.Climate action	23,920	13.Climate action	9121
15.Life on land	8660	15.Life on land	3325
14.Life below water	6037	14.Life below water	2301
02.Zero hunger	5253	02.Zero hunger	2009
05.Gender equality	4897	05.Gender equality	808
06.Clean water and sanitation	989	06.Clean water and sanitation	418
12.Responsible consumption and production	933	12.Responsible consumption and production	386
11.Sustainable cities and communities	607	11.Sustainable cities and communities	239
07.Affordable and clean energy	336	07.Affordable and clean energy	208
09.Industry innovation and infrastructure	192	09.Industry innovation and infrastructure	134
10.Reduced inequality	149	10.Reduced inequality	76
01.No poverty	136	01.No poverty	75
04.Quality education	110	04.Quality education	29
08.Decent work and economic gowth	69	08.Decent work and economic gowth	27
16.Peace and justice strong institutions	62	16.Peace and justice strong institutions	25
17.Partnerships for the goals	29	17.Partnerships for the goals	21

Figure S10. Column plot of the yearly proportion (%) of papers related to sheep or goats published from 1970 to 2004 in accordance with the Sustainable Development Goals served in the papers.

(a) Papers related to sheep

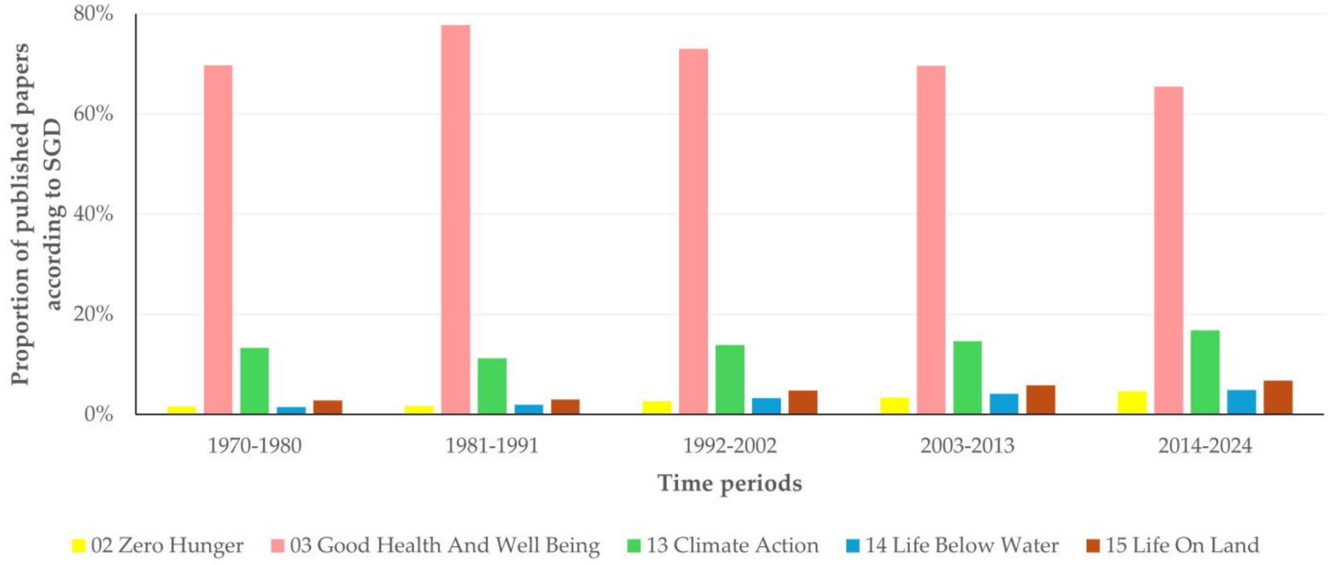

(b) Papers related to goats

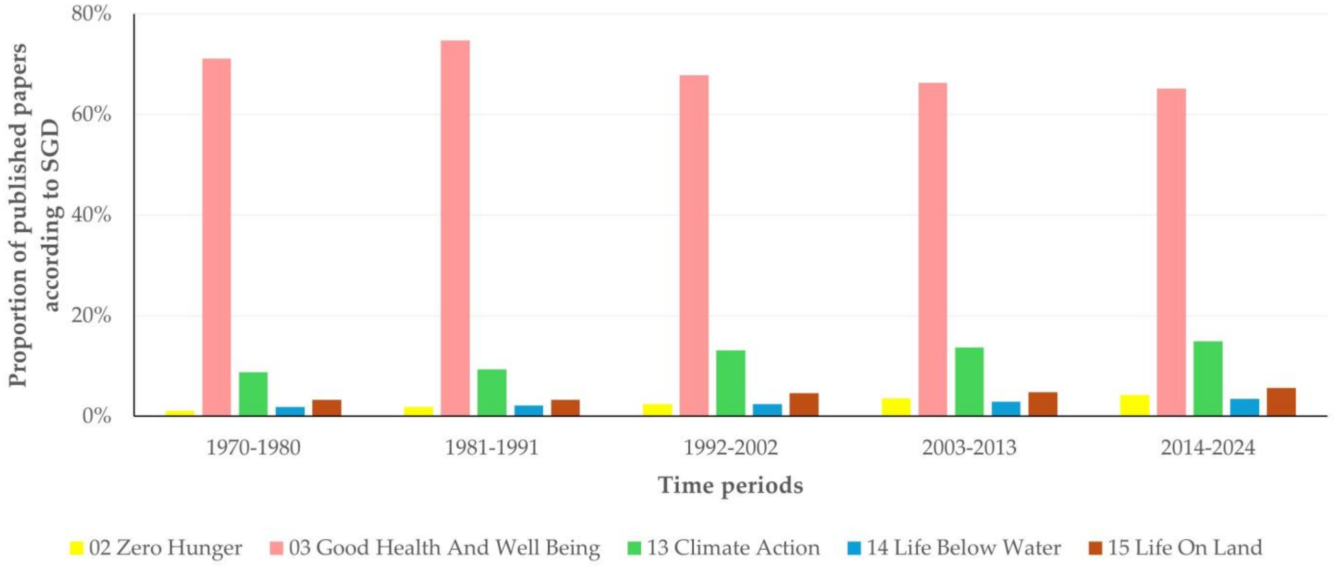

Figure S11. Diagrams of joint authorships among the ten authors with most papers on sheep (a) or goats (b) published from 1970 to 2024 (letters within the circles denote authors, but do not correspond to their surnames in order to guarantee private data protection).

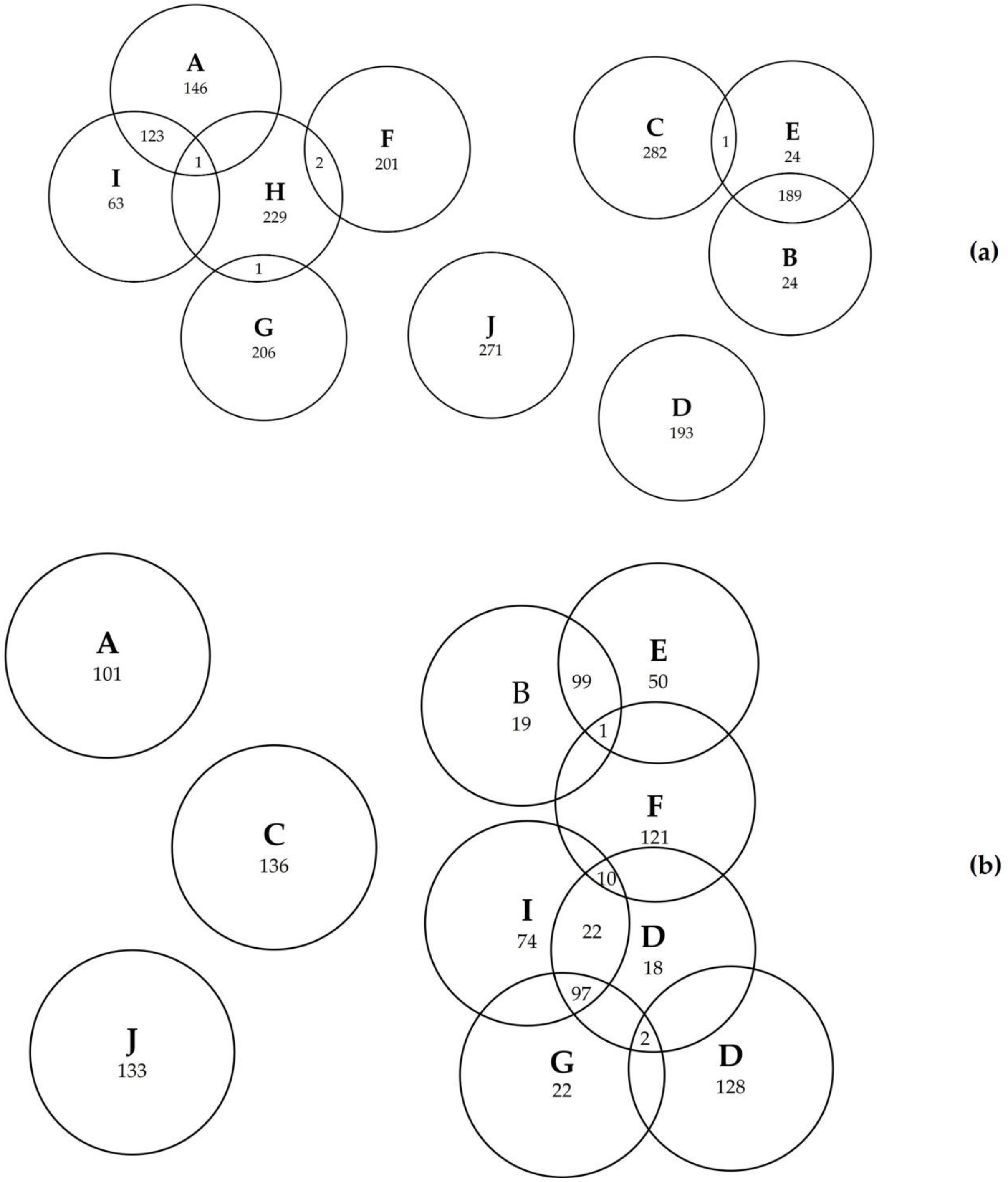

Supplement: Supplementary file 1 [file animals-16-01163-s001.zip › animals-4192603-supplementary.pdf]
